# CXCR1^+^ neutrophil infiltration orchestrates response to third-generation EGFR-TKI in EGFR mutant non-small-cell lung cancer

**DOI:** 10.1038/s41392-024-02045-2

**Published:** 2024-12-06

**Authors:** Haowei Wang, Anwen Xiong, Xiaoxia Chen, Junhong Guo, Zhuoran Tang, Chunyan Wu, Shengxiang Ren, Caicun Zhou, Jian Chen, Likun Hou, Tao Jiang

**Affiliations:** 1 Department of Medical Oncology, Shanghai Pulmonary Hospital & Thoracic Cancer Institute, Tongji University School of Medicine, Shanghai, China; 2 Department of Medical Oncology, Shanghai East Hospital, Tongji University School of Medicine, Shanghai, China; 3 Department of Pathology, Shanghai Pulmonary Hospital, Tongji University School of Medicine, Shanghai, China; 4 Department of Thoracic Surgery, Shanghai Pulmonary Hospital, Tongji University School of Medicine, Shanghai, China

**Keywords:** Lung cancer, Tumour biomarkers, Cancer microenvironment

## Abstract

Although third-generation Epidermal growth factor receptor—tyrosine kinase inhibitors (EGFR-TKI) is standard of care for patients with EGFR-mutant Non-small cell lung cancer (NSCLC), little is known about the predictors of response or resistance. Here, we integrated single-cell RNA (scRNA) sequencing, bulk RNA sequencing, multiplexed immunofluorescence and flow cytometry data from pretreatment and post-resistant tumor samples of EGFR-mutant NSCLC patients received third-generation EGFR-TKIs. We show that resistant samples had a markedly enriched CXCR1^+^ neutrophils infiltration (*P* < 0.01) than pretreatment samples, which were distinguished from other subtypes of neutrophils and displayed immunosupressive characteristics. Spatial analysis showed that increased CXCR1^+^ neutrophils predominantly infiltrated into the tumor core in resistant samples and the average distance of neutrophils to tumor cells markedly reduced from 33 to 19 μm. Deep analysis of scRNA and bulk RNA sequencing data revealed the increased interactions between CXCR1^+^ neutrophils and tumor cells and activated TNF-α/NF-κB signaling pathway in tumor cells of resistant samples. In vitro and in vivo experiments validated that CXCR1^+^ neutrophils resulted in resistance to third-generation EGFR-TKI via activating TNF-α/NF-κB signaling pathway in tumor cells. Importantly, patients with low pretreatment CXCR1^+^ neutrophil infiltration abundance had a dramatically longer progression-free survival (11.8 vs. 7.5 months; *P* = 0.019) and overall survival (33.0 vs. 23.5 months; *P* = 0.029) than those with high infiltration abundance. Collectively, these findings suggest that CXCR1^+^ neutrophils infiltration was associated with the efficacy of third-generation EGFR-TKI in patients with EGFR-mutant NSCLC.

## Introduction

Non-small-cell lung cancer (NSCLC), which constitutes the predominant histological subtype of lung malignancies, accounts for approximately 85% of total lung cancer cases.^1,2^ Despite significant advances in systemic treatments over the past decade, NSCLC still signifies a leading causing to global cancer-related mortality.^3^ Among these various treatment strategies available, molecular targeted therapies, specifically tyrosine kinase inhibitors (TKIs), have become the standard first-line treatment for advanced NSCLC cases with driver gene alterations. These agents target aberrant signaling pathways critical for tumor growth and survival. Notable targets include the epidermal growth factor receptor (EGFR), anaplastic lymphoma kinase (ALK), ROS proto-oncogene 1 receptor tyrosine kinase (ROS1), ret proto-oncogene (RET), and b-raf proto-oncogene serine/threonine kinase (BRAF) V600E.^4^ Patients with these specific genetic alterations have experienced higher objective response rates (ORR), prolonged progression-free survival (PFS), overall survival (OS) and improved quality of life compared to traditional treatment.^5–7^

EGFR mutation is one of the most common oncogenic alterations in advanced NSCLC, particularly prevalent in non-smokers and East Asian populations.^8,9^ Several elegant phase II/III trials have demonstrated that third-generation EGFR-TKIs showed superior efficacy than first-generation EGFR-TKIs as first-line setting in patients with EGFR-mutant NSCLC.^10–13^ Additionally, the third-generation EGFR-TKIs have also displayed greater efficacy than conventional chemotherapy in second or above-line setting for patients with acquired EGFR T790M mutation after first- and second-generation EGFR-TKIs treatment.^14–17^ In our previous studies, we reported a novel third-generation EGFR-TKI, SH-1028, which displayed approximately 198-fold greater sensitivity compared to its wild-type counterparts.^18^ In the key phase II trial, SH-1028 showed a satisfying efficacy and tolerable toxicity, with an objective response rate (ORR) of 60.4% and a median progression-free survival (PFS) of 12.6 months.^19^ These encouraging results set the stage for further clinical investigations. Recently, SH-1028 was evaluated in a phase III trial (NCT04239833), comparing its efficacy and safety to first- and second-generation EGFR-TKIs as a first-line treatment in advanced EGFR-mutant NSCLC. The trial results were compelling, with SH-1028 demonstrating a dramatically longer PFS (median PFS: 19.3 months) compared to the comparator group (median PFS: 9.8 months). Based on these findings, SH-1028 has been approved by China’s National Medical Products Administration (NMPA) as a first-line therapy for patients with advanced EGFR-mutant NSCLC. Although third-generation EGFR-TKIs have become the standard of care for patients with EGFR-mutant NSCLC, not all of patients could benefit from them. The inevitability of resistance remains a substantial concern.^20^ Currently, reported resistance mechanisms could be categorized into three major groups: secondary EGFR alterations, activation of bypass signaling pathways, and phenotypic or histological transformations.^21,22^ However, the detailed characterization of these mechanisms is hitherto poorly defined and the identification of robust markers for predicting treatment efficacy of third-generation EGFR-TKIs warrant further investigation.

Immunotherapy targeting PD-1 or PD-L1 (PD-1 blockade) alone or plus cytotoxic chemotherapy have achieved huge success in the treatment of patients with advanced NSCLC.^23–26^ These therapies enhance antitumor immune responses by blocking inhibitory signals that restrain T-cell activation. However, their effectiveness in EGFR-mutant NSCLC has been limited. The application of PD-1 blockade, alone or combined with chemotherapy or EGFR-TKIs after progression on EGFR-TKI treatments, has not yielded substantial clinical benefits.^27,28^ Initial explanations ascribed this poor response to the cold tumor immune microenvironment (TME) characteristics of EGFR-mutant lung tumors, including low immunogenicity, heterogeneity of PD-L1 expression, few infiltrations of cytotoxic T cells, and so on.^29–33^ Tumor cells can recruit immune and stromal cells, manipulating their functions to establish an immunosuppressive environment that shields them from immune-mediated elimination. Conversely, the tumor microenvironment can influence tumor cell biology and alter drug sensitivity. Therefore, the dynamic interactions between tumor cells and their surrounding cells can impact their response to therapy. A recent study further reported no significant change in anti-tumor cell infiltration or cytotoxicity between pretreatment and post-TKI-resistant samples.^34^ Due to the limited studies with limited sample size on TKI-induced tumor microenvironment remodeling, the immunological effects of EGFR-TKIs in treatment-responsive tumors and dynamic changes of tumor immune microenvironment features before and after resistance to EGFR-TKIs remain largely undetermined.

To investigate these issues, a comprehensive investigation into biological parameters parameters that could predict the efficacy of third-generation EGFR-TKIs was conducted. We also aimed to elucidate the dynamic changes and functions within the tumor immune microenvironment associated with resistance to TKIs in EGFR-mutant NSCLC. Single cell RNA (scRNA) sequencing, bulk RNA sequencing, multiplexed immunofluorescence (mIF) and flow cytometry were performed on pretreatment and post-resistant tumor samples from EGFR-mutant NSCLC patients received third-generation EGFR-TKIs. I*n vitro* and in vivo experiments were also conducted to validate the results from clinical tumor samples. Our findings together suggest that CXCR1^+^ neutrophil infiltration and its dynamic changes are associated with the efficacy of third-generation EGFR-TKI in patients with EGFR-mutant NSCLC. The presence of CXCR1^+^ neutrophils contribute to the development of resistance by interacting with tumor cells. This association highlights the potential of CXCR1^+^ neutrophil infiltration as a predictive biomarker for treatment response and as a therapeutic target to overcome resistance to EGFR-TKI therapy.

## Results

### Baseline features of included patients

**Fig. 1:**
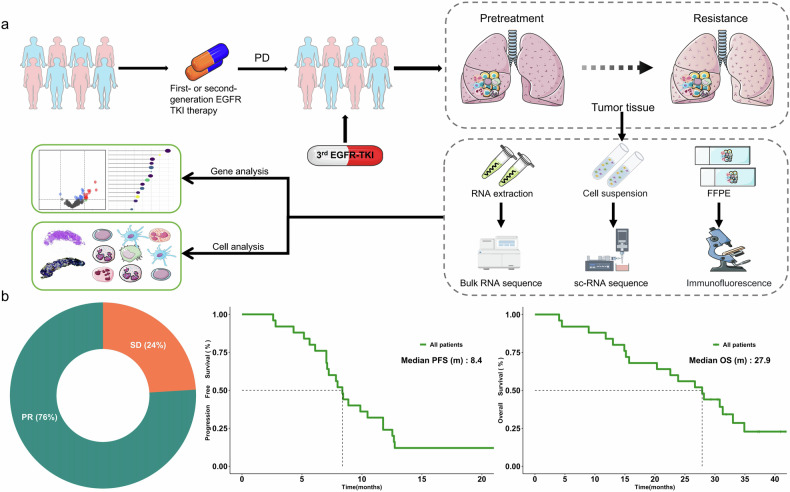
The study design and patients’ clinical outcomes. **a** Flowchart of patient’s enrollment, sample collection and multiple omic analysis (the graphical model figure was created using Figdraw). **b** Objective response rate, progression-free survival and overall survival of include patients. FFPE, formalin fixed paraffin-embedded; PR, partial response; SD, stable disease; PFS, progression-free survival; OS, overall survival

This study firstly incorporated 25 patients with EGFR T790M mutant NSCLC received third-generation EGFR-TKI from a phase II trial (NCT03823807, Fig. 1a). The median age was 64 years, ranging from 43 to 78 years. The majority of them (60%) were male, and 68% were identified as either current or former smokers. Pathological confirmation of adenocarcinoma was observed in all patients, with a significant majority (84%) presenting with stage IV disease. Only a patient possessed the EGFR T790M mutation exclusively, whereas the rest had additional EGFR subtype mutations, predominantly EGFR exon 19 deletions (52%). The presence of metastasis was noted in some cases, with brain (*n* = 7) or bone (*n* = 8) being the most common sites (Supplementary Table 1). The overall ORR was 76% (Fig. 1b). During a follow-up period of 41.9 months, the median PFS and OS were 8.4 and 27.9 months, respectively (Fig. 1b).

### Resistant samples possess an immunosuppressive microenvironment and higher neutrophil infiltration

**Fig. 2:**
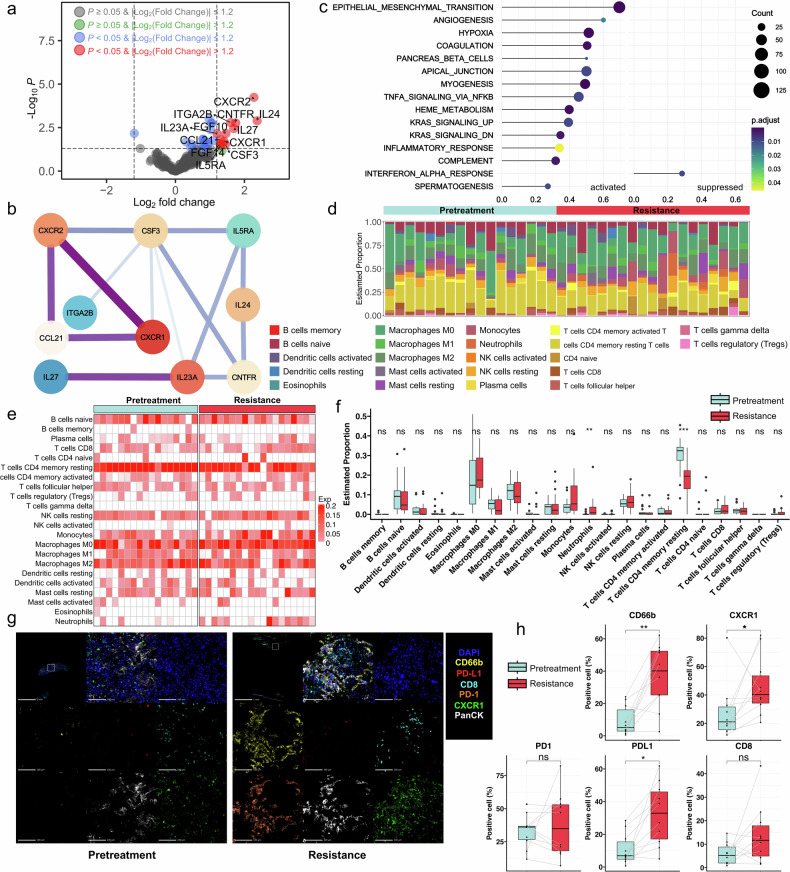
Transcriptomic and multiplexed immunofluorescence analyses on paired pretreatment and post-resistant tumor samples. **a** Volcano plots showing the expression profiles of cytokines. **b** Protein interaction mapping of differentially expressed cytokines. **c** GSEA enrichment analysis with hallmarker gene sets. **d** Proportion comparison of 22 immune cells infiltration abundance between pretreatment and resistant samples. **e** Heatmap of 22 immune cells in pretreatment and resistant samples. **f** Comparisons of 22 immune cells infiltration abundance between pretreatment and resistant samples. **g** Multi-color immunofluorescence staining of CD66b^+^, CD8^+^, PD-1^+^, PD-L1^+^, CXCR1^+^ and PanCK^+^ cells between pretreatment and resistant samples. **h** Paired comparison of CD66b, CD8, PD-1, PD-L1, CXCR1 and PanCK expression between pretreatment and resistant samples. ns, *P* > 0.05; *P < 0.05; **P < 0.01; ***P < 0.001

To elucidate the dynamic changes of tumor immune microenvironment features after drug resistance, we firstly performed bulk RNA sequencing on the available high-quality samples. After strict quality control and selection, 17 pretreatment and 19 posttreatment samples were identified (17 were paired samples and their baseline features were listed in Supplementary Table 1). In the resistant samples, a significant upregulation of 856 genes and downregulation of 53 genes were observed. Within these differential expressed genes (DEGs), a total of 208 immune-associated genes were brought to light (Fig. 2a). 12 of these genes showed an increased expression, key among them being CXCR1, CXCR2, and CSF3, which play vital role in neutrophils’ functionality and lifecycle (Fig. 2b). To demystify the biological pathway alterations, we performed enrichment analyses based on Gene Ontology (GO) and Kyoto Encyclopedia of Genes and Genomes (KEGG). These DEGs prominently displayed functions such as bacterial defense response, plasma-membrane mediated cell-cell adhesion, myeloid leukocyte activation, and humoral immune response (Supplementary Fig. 1a). KEGG pathway analysis disclosed an upregulation of several pathways during drug resistance, including neuroactive ligand-receptor interaction, JAK-STAT signaling pathway, cytokine-cytokine receptor interaction, and complement and coagulation cascades among others. However, only a few pathways were found enriched with downregulated genes (Supplementary Fig. 1b). Further, gene set enrichment analysis (GSEA) also displayed several upregulation of the hallmark gene sets related to tumor progression and EGFR-TKI resistance, including epithelial-mesenchymal transition, angiogenesis, hypoxia, and TNF-α signaling via NF-κB. Concurrently, we observed a downregulation of interferon alpha response, together painting a picture of an immunosuppressive microenvironment (Fig. 2c).

Using CIBERSORT, we examined the differences of immune infiltrations between pretreatment and resistant samples. The proportions of 22 immune cell types varied considerably (Fig. 2d, e). Notably, we observed a significant increased neutrophil infiltration in resistant samples (*P* = 0.004), together with a significant reduction in resting CD4 memory T cell infiltration (*P* < 0.001; Fig. 2f and Supplementary Fig. 2). These results were confirmed by using Xcell, reinforcing the increased neutrophil infiltration in resistant samples (*P* = 0.048; Supplementary Fig. 3a).

To validate the increased neutrophils infiltration in the resistant samples, we conducted Hematoxylin and Eosin (HE) staining to select high-quality samples for mIF staining (Fig. 2g). Ten of them with both adequate pretreatment and resistant tissue samples were identified as suitable for mIF analysis. There was a significant increase in neutrophils (*P* < 0.01) and CXCR1 expressing cells (*P* < 0.05) in resistant samples than pretreatment samples. A notable increase in PD-L1 expression level was also detected in resistant samples (*P* < 0.05), further indicating the emergence of an immunosuppressive tumor microenvironment in resistant tumors (Fig. 2h).

### scRNA sequencing analysis reveals CXCR1^+^ neutrophils are enriched in resistant samples

**Fig. 3:**
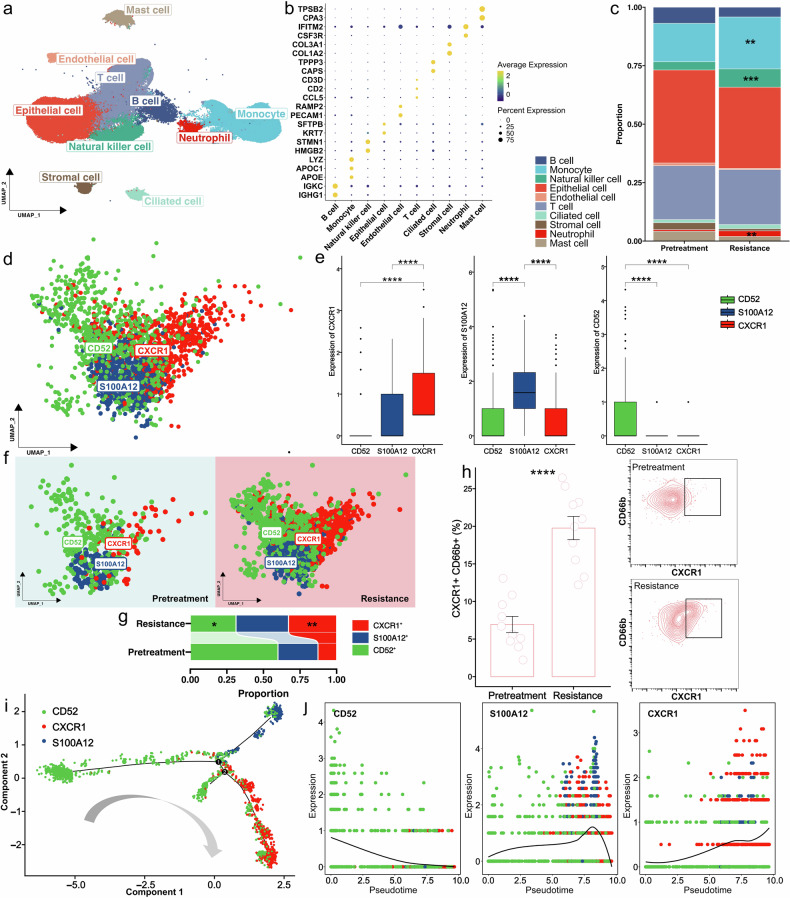
Single cell analysis reveal neutrophil atlas in pretreatment and resistant samples. **a** The UMAP plot of all celltypes. **b** Marker genes were used to annotated these cell groups. **c** The proportion of each cell in pretreatment and resistance. **d** The UMAP plot of neutrophils. **e** High expressed genes were used to delimit three neutrophil subgroups. **f** The UMAP plot of neutrophils in pretreatment and resistance. **g** The proportion of each subgroup in pretreatment and resistance. **h** Flow cytometry plots of CXCR1^+^ neutrophil change in pretreatment and resistance. **i** Unsupervised transcriptional trajectory of neutrophil from Monocle. **j** Expression of marker genes with pseudotime based on transcriptional trajectory. ns, *P* > 0.05; *P < 0.05; **P < 0.01; ***P < 0.001

To further investigate the role of neutrophils infiltration in mediating response or resistance to EGFR-TKIs, we conducted scRNA sequencing on 34 samples (22 pretreatment and 12 resistant) from 22 EGFR-mutant NSCLC patients received third-generation EGFR-TKIs in a real-world cohort. Baseline parameters were provided in Supplementary Table 2. After quality control, 141,292 cells were analyzed. Unsupervised clustering identified 10 distinct cell clusters (Fig. 3a), including B cells, monocytes, natural killer cells, epithelial cells, endothelial cells, T cells, ciliated cells, stromal cells, neutrophils, and mast cells based on canonical marker genes (Fig. 3b). We firstly compared the cellular composition of each cell type between pretreatment and resistant samples. Significant increases were observed in the proportions of neutrophils (*P* < 0.01), monocytes (*P* < 0.01), and natural killer cells (*P* < 0.001) in resistant samples (Fig. 3c). Further analysis of neutrophil heterogeneity involved dimensionality reduction and clustering of 2,338 neutrophils, identifying three subclusters (CD52^+^, S100A12^+^, and CXCR1^+^) based on differential gene expression (Fig. 3d, e). Notably, the proportion of CXCR1^+^ neutrophils significantly increased (*P* < 0.01), while CD52^+^ neutrophils markedly decreased (*P* < 0.05) in resistant samples compared to pretreatment samples (Fig. 3f, g). Flow cytometry confirmed a significant increase in CXCR1^+^ neutrophils in resistant samples (*P* < 0.001; Fig. 3h and Supplementary Fig. 4). Pseudotime analysis delineated differentiation trajectories for these subpopulations (Fig. 3i), and ranking by pseudotime suggested that CD52^+^ neutrophils initiate and subsequently diverge towards S100A12^+^ and CXCR1^+^ subtypes (Fig. 3j and Supplementary Fig. 5).

### Delineating the role of CXCR1^+^ neutrophils in resistance to EGFR-TKIs

**Fig. 4:**
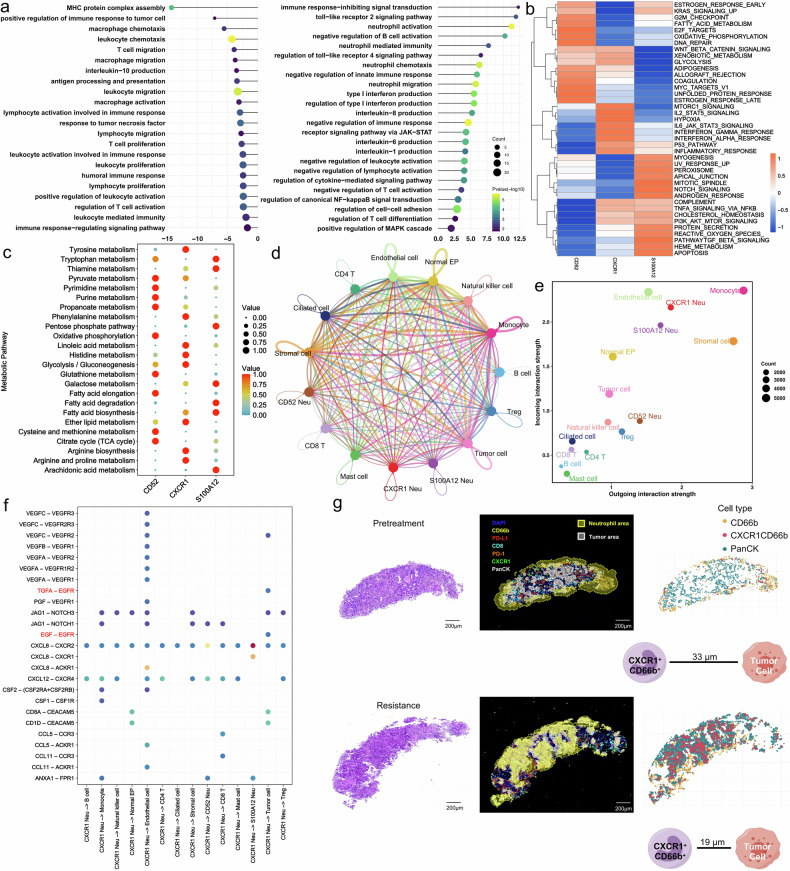
CXCR1^+^ neutrophils exhibit immunosuppressive characteristics and play vital role in tumor microevironment. **a** Downregulated and upregulated pathway of CXCR1^+^ neutrophils. **b** The heatmap of hallmarker gene sets using GSVA. **c** Metabolism pathway activity of neutrophil subsets determined by scMetabolism. **d** Cell communications detected by CellChat analysis. **e** The outgoing and incoming interaction strength of each cell type. **f** The ligand-receptor pairs between CXCR1^+^ neutrophils and tumor cells. **g** Spatial analysis of CXCR1^+^ neutrophils between pretreatment and resistant samples

To investigate the role of CXCR1^+^ neutrophils in resistance to EGFR-TKIs, we compared the cytokine profiles of different neutrophil subsets (Supplementary Fig. 6a). CD52^+^ neutrophils exhibited high CCL5 expression, which is known to recruit T cells.^35^ In contrast, CXCR1^+^ neutrophils demonstrated high levels of PD-L1 (CD274), suggesting an immunosuppressive role. Distinctively, only CD52^+^ neutrophils exhibited enrichment of MHC class II molecules, such as HLA-DQB1 and HLA-DRA, whereas CXCR1^+^ and S100A12^+^ subsets primarily expressed MHC class I molecules, including HLA-B and HLA-C (Supplementary Fig. 6b). To understand how pathway activity differs among tumor-associated neutrophil subsets, we analyzed the pathway variance and found significant downregulation of pathways that positively regulate immune cell functions, such as T cell proliferation, migration, and activation in CXCR1^+^ neutrophils (Fig. 4a). Conversely, pathways related to neutrophil activation, migration, and chemotaxis were upregulated (Fig. 4a). Key signaling pathways, such as NF-κB and JAK-STAT, were also found to be upregulated in CXCR1^+^ neutrophils (Fig. 4b), as confirmed by KEGG analysis (Supplementary Fig. 6c). Additionally, neutrophil extracellular trap formation (Supplementary Fig. 6c), which can promote tumor proliferation and metastasis, was increased in CXCR1^+^ neutrophils.^36,37^ Gene Set Variation Analysis (GSVA) of hallmark gene sets revealed distinct pathway profiles among the three subsets, with CXCR1^+^ neutrophils exhibiting characteristics related to hypoxia, MTORC1, IL-2 and IL-6 signaling. Metabolic pathway activity in the neutrophil subsets was also quantified (Fig. 4c), showing significant enrichment of tyrosine, phenylalanine metabolism, and arginine biosynthesis in CXCR1^+^ neutrophils. Meanwhile, CD52^+^ neutrophils primarily activated arachidonic acid metabolism, glutathione metabolism, and tryptophan metabolism, while the pentose phosphate pathway and fatty acid biosynthesis and degradation were predominant in S100A12^+^ neutrophils (Fig. 4c).

Considering the significant increase of CXCR1^+^ neutrophils in resistant samples, we then conducted CellChat analysis to elucidate the role of CXCR1^+^ neutrophils within the tumor microenvironment. Given the heterogeneity among epithelial and T cells, these groups were further subdivided and annotated. Epithelial cells were categorized as normal epithelial (Normal-EP) and tumor cells using the CopyKAT algorithm (Supplementary Fig. 7a, b), and T cells were classified as CD8, CD4, and Treg based on gene markers (Supplementary Fig. 7c, d). CellChat analysis revealed significant communication between various cell types (Fig. 4d and Supplementary Fig. 8), particularly noting that CXCR1^+^ neutrophils demonstrated high interaction strength in both outgoing and incoming signals (Fig. 4e). Analysis of ligand-receptor pairs revealed interactions between CXCR1^+^ neutrophils and tumor cells involving TGFA-EGFR and EGF-EGFR, potentially explaining aspects of resistance to third-generation EGFR-TKIs due to sustained EGFR activation (Fig. 4f). Moreover, the strength of cell interactions was higher in resistant samples (Supplementary Fig. 9a), with communication patterns showing various degrees of regulation (Supplementary Fig. 9b). After resistance, CXCR1^+^ neutrophils communicated more effectively with tumor cells, regardless of the direction of the signaling (Supplementary Fig. 9c). Additionally, the role of CXCR1^+^ neutrophils in cellular communication became more pronounced in resistant samples compared to pretreatment samples (Supplementary Fig. 9d, e). All pathways enriched in CellChat were analyzed, revealing significant changes in cellular communication pathways after developing drug resistance, with pathways including EGF, IL-2, and VISTA significantly activated (Supplementary Fig. 10). Furthermore, ligand-receptor pairs related to EGFR between CXCR1^+^ neutrophils and tumor cells were significantly increased in resistance and communication with Treg cells also increased (Supplementary Fig. 11). Lastly, we conducted a density map of neutrophil distribution and their spatial proximity to tumor areas. The results showed that neutrophils predominantly surrounded the tumor area before treatment but infiltrated into the tumor core in resistant samples. The average distance of CXCR1^+^ neutrophils to tumor cells significantly decreased from 33 μm in pretreatment samples to 19 μm in resistant samples (Fig. 4g).

### Early disease progression group shows an immunosuppressive microenvironment with higher CXCR1^+^ neutrophil infiltration

**Fig. 5:**
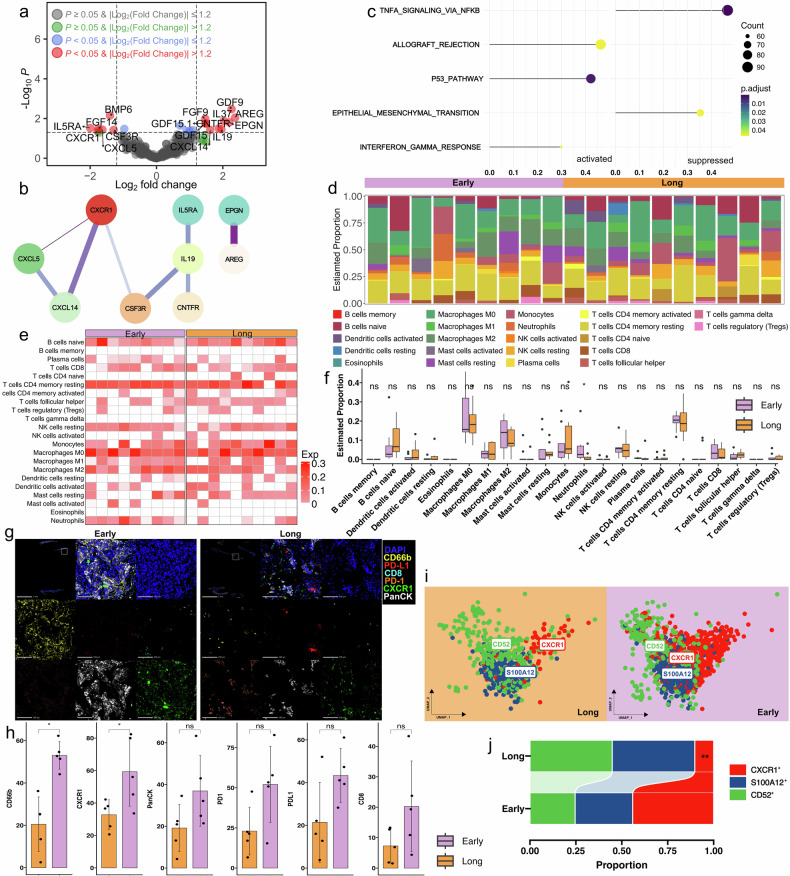
Transcriptomic and multiplexed immunofluorescence analyses on samples from long benefit versus early progression groups. **a** Volcano plots showing the expression profiles of cytokines. **b** Protein interaction mapping of differentially expressed cytokine. **c** GSEA enrichment analysis with Hallmarker gene sets. **d** Proportion comparison of 22 immune cells infiltration abundance between long benefit and early progression groups. **e** Heatmap of 22 immune cells in long benefit and early progression groups. **f** Comparisons of 22 immune cells infiltration abundance between long benefit and early progression groups. **g** Multi-color immunofluorescence staining of CD66b^+^, CD8^+^, PD-1^+^, PD-L1^+^, CXCR1^+^ and PanCK^+^ cells between long benefit and early progression groups. **h** The comparisons of CD66b, CD8, PD-1, PD-L1, CXCR1 and PanCK expression between long benefit and early progression groups. **i** The umap plot of neutrophil between long benefit and early progression groups. **j** The proportion of each subgroup between long benefit and early progression groups. ns, *P* > 0.05; *P < 0.05; **P < 0.01; ***P < 0.001

Using median PFS as the cutoff, we categorized 19 patients with high-quality samples into two cohorts, namely the ‘long benefit’ and ‘early disease progression’ groups. Within the long benefit group, we detected an upregulation of 653 genes and downregulation of 431 genes. Among these DEGs, CXCR1 was significantly downregulated in the long benefit group compared to the early disease progression (*P* = 0.045; Fig. 5a). The STRING analysis suggested a tight interplay between CXCR1 and proteins expressed by other differential genes (Fig. 5b), hinting at a possible role of CXCR1 in the inferior treatment outcomes. GO analyses showed the enrichment of signaling receptor activator activity, extracellular matrix structural constituent, growth factor activity, and collagen-containing extracellular matrix in the long benefit group (Supplementary Fig. 12a). KEGG pathway analysis showed upregulation in cytokine-cytokine receptor interaction, downregulation in chemokine signaling pathway, hematopoietic cell lineage, etc. in the long benefit group (Supplementary Fig. 12b). Compared with the early disease progression group, GSEA of hallmarker gene sets showed that p53 pathway, and interferon-gamma response were upregulated in the long benefit group, indicating an activated intratumor immunity. Conversely, the TNF-α signaling via NF-κB and epithelial-mesenchymal transition pathway was upregulated in early progression group, which were previously reported associated with EGFR-TKI resistance (Fig. 5c).

Next, we compared the difference of immune cell infiltration abundance between long benefit and early progression groups (Fig. 5d, e). CIBERSORT analysis showed samples in early progression group had a dramatically higher neutrophil infiltration level than the long benefit group (*P* = 0.029), while other immune cells infiltration abundance was similar (Fig. 5f and Supplementary Fig. 13). This finding was corroborated by Xcell analysis, which confirmed higher neutrophil infiltration in the early progression group (*P* = 0.037; Supplementary Fig. 3b). mIF analysis revealed that samples in the early progression group had markedly higher counts of neutrophils (*P* < 0.05) and CXCR1-expressing cells (*P* < 0.05) than those in the long benefit group. PD-L1, PD-1 and CD8 expression levels were comparable between two groups (Fig. 5g, h). Single cell anaylsis varified the increase of CXCR1^+^ neutrophil in early progression group (*P* < 0.01) while no difference was found in CD52^+^ and S100A12^+^ subgroups (Fig. 5i, j).

### CXCR1^+^ neutrophils mediate resistance to third-generation EGFR TKIs via TNF-α/NF-κB

**Fig. 6:**
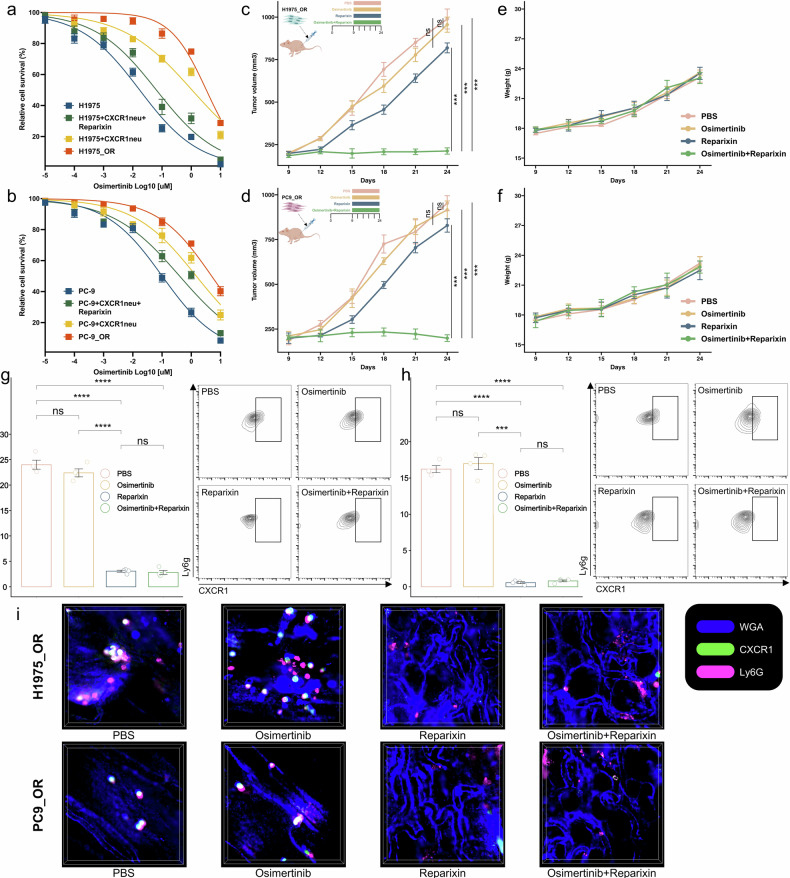
CXCR1^+^ neutrophils mediate resistance to Third-Generation EGFR TKIs. **a** Relative survival curve describing the viability of indicated H1975 treated with the indicated concentrations of osimertinib. **b** Relative survival curve describing the viability of indicated PC-9 treated with the indicated concentrations of osimertinib. **c** Growth curves of xenograft tumors derived from H1975_OR following osimertinib treatment or in combination with reparixin. **d** Growth curves of xenograft tumors derived from PC-9_OR following osimertinib treatment or in combination with reparixin. **e** Body weight changes during experiment in H1975_OR mouse models. **f** Body weight changes during experiment in PC-9_OR mouse models. **g** Flow cytometry of CXCR1^+^ neutrophil proportion in H1975_OR mouse models. **h** Flow cytometry of CXCR1^+^ neutrophil proportion in PC-9_OR mouse models. **i** Represent images of intravital tumor imaging with CXCR1^+^ neutrophils by DAOSLIMIT in the H1975_OR and PC-9_OR mouse models. OR, osimertinib resistance

Integration of previous multi-omic data revealed that CXCR1^+^ neutrophil could result in the inferior response to third-generation EGFR-TKIs via TNF-α/NF-κB signaling pathway (Figs. 2c, 4a, b and 5c). To validate this, we performed in vitro and in vivo experiments using two well-known EGFR-mutant cell lines (H1975 and PC-9) and their osimertinib-resistant cell lines (H1975_OR and PC-9_OR). These four cell lines were extensively used in our previous studies.^38–40^ In vitro experiments showed that co-culture of CXCR1^+^ neutrophils with H1975 or PC-9 significantly reduced sensitivity to osimertinib (IC_50_: 0.017 vs. 1.064 μM, *P* < 0.05; 0.012 vs. 1.491 μM, *P* < 0.05; Fig. 6a, b). The addition of reparixin (a non-competitive allosteric blocker of CXCR1 and CXCR2) in the co-culture system markedly reversed the resistant phenotype (Fig. 6a, b). Subsequently, we leveraged H1975_OR and PC-9_OR cell lines to establish EGFR-mutant NSCLC xenografts. The results showed that osimertinib or reparixin monotherapy cannot significantly reduce tumor growth, but osimertinib plus reparixin could significantly inhibit tumor growth without effect on body weight (Fig. 6c, d, e, f and Supplementary Fig. 14). Flow cytometry and intravital imaging of tumors by DAOSLIMIT showed that reparixin significantly reduced the intratumoral infiltration of CXCR1^+^ neutrophils (Fig. 6g, h, i and Supplementary Fig. 15), irrespective of whether in combination with osimertinib. The live imaging techniques were employed to monitor the dynamics of CXCR1^+^ neutrophils within the live tumors, revealing substantial infiltration through tumor vasculature, which was effectively reduced by reparixin (Supplementary Movie 1 and 2).

**Fig. 7:**
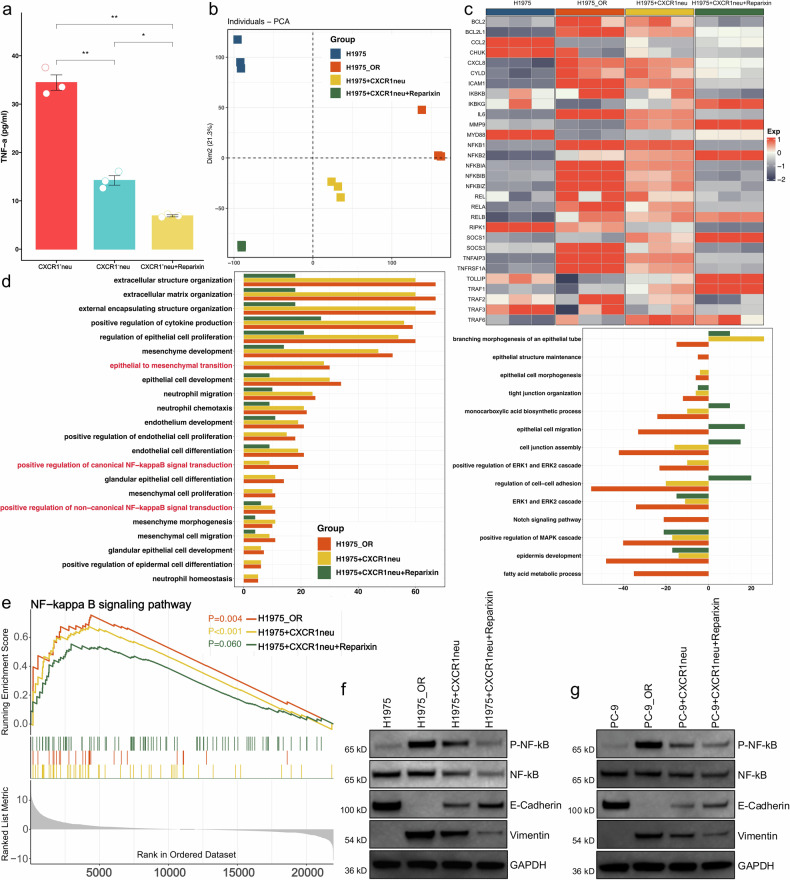
CXCR1^+^ neutrophils secrete TNF-α to activate NF-κB and EMT pathways in tumor cells. **a** TNF-α level in supernatants from CXCR1^+^ and CXCR1^-^ neutrophils. **b** PCA analysis. **c** Heatmap analysis of NF-κB related key gene expression. **d** GO analysis of different expressed genes. **e** GSEA enrichment analysis of NF-κB signaling pathway. **f** Representative western blot images of P-NF-κB, NF-κB, E-Cadherin, Vimentin, GAPDH protein expression in H1975 and H1975_OR groups. **g** Representative western blot images of P-NF-κB, NF-κB, E-Cadherin, Vimentin, GAPDH protein expression in PC-9 and PC-9_OR groups. GAPDH, Glyceraldehyde-3-phosphate dehydrogenase; OR, osimertinib resistance

To detect the changes of TNF-α/NF-κB signaling pathway before and after treatment, we firstly measured cytokine levels using ELISA in the supernatants from different treatment groups. The increased TNF-α secretion by CXCR1^+^ neutrophils was observed, which was significantly inhibited by reparixin (*P* < 0.01; Fig. 7a). Bulk RNA sequencing of tumor cells post co-culture revealed significant changes in gene expression profiles (Fig. 7b). Heatmap analysis of NF-κB signaling pathway-related key genes showed that tumor cells co-cultured with CXCR1^+^ neutrophils exhibited gene expression patterns similar to resistant group, which reverted to unprocessed upon reparixin treatment (Fig. 7c). Pathway enrichment analysis of differential genes showed significant upregulation of NF-κB and EMT pathways in tumor cells co-cultured with CXCR1^+^ neutrophils, which was notably reduced in reparixin treatment group (Fig. 7d). GSEA further confirmed that reparixin effectively inhibited the activation of NF-κB pathways induced by CXCR1^+^ neutrophils (Fig. 7e). Western blot analysis also showed the increased phosphorylated NF-κB and Vimentin expression and decreased E-cadherin expression in tumor cells co-cultured with CXCR1^+^ neutrophils, which reversed upon addition of reparixin (Fig. 7f, g). RNA-seq analysis using EGFR-mutant NSCLC xenografts also demonstrated the similar changes of TNF-α/NF-κB signaling pathway and EMT-related markers before and after treatment (Supplementary Fig. 16).

### Baseline CXCR1^+^ neutrophil infiltration level is associated with clinical outcomes of patients receiving third-generation EGFR-TKI

**Fig. 8:**
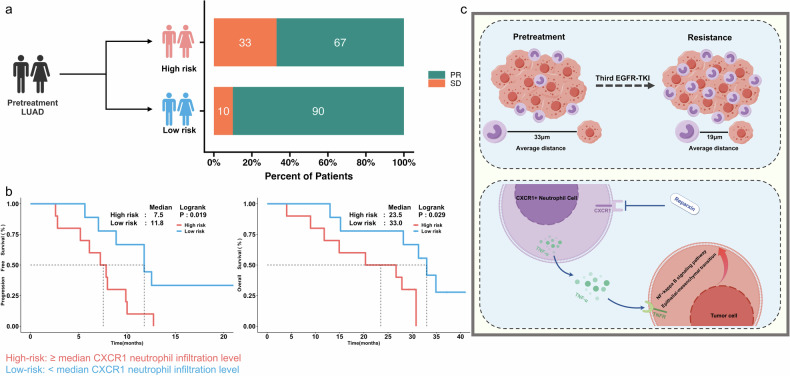
Comparison of treatment outcomes between patients with high and low baseline CXCR1^+^ neutrophil infiltration level. **a** Comparison of objective response rate between patients with high (high risk) and low (low risk) baseline CXCR1^+^ neutrophil infiltration level. **b** Comparison of progression-free survival (left) and overall survival (right) between patients with high risk and low risk group. **c** Graphical abstract of the study (the graphical abstract was created by Figdraw). PR, partial response; SD, stable disease; PFS, progression-free survival; OS, overall survival

In order to evaluate whether neutrophil infiltration predicts the response to third-generation EGFR-TKIs, we then stratified patients into high-risk (pretreatment tumors samples with ≥ median CXCR1^+^ neutrophil infiltration level) and low-risk (pretreatment tumors samples with < median infiltration level) groups using the median CXCR1^+^ neutrophil infiltration level in pre-treatment samples as cutoff. The low-risk group exhibited a superior ORR compared to the high-risk group (90% vs. 67%; *P* = 0.303; Fig. 8a) but it did not reach statistical significance due to very limited sample size. The median PFS in low-risk group was significantly prolonged in comparison to the high-risk group (median PFS: 11.8 vs. 7.5 months; *P* = 0.019; Fig. 8b). Moreover, compared with high-risk group, patients in the low-risk group had a markedly better OS (median OS: 33.0 vs. 23.5 months; *P* = 0.029; Fig. 8b). The multivariate analyses incorporating clinicopathological features showed that baseline CXCR1^+^ neutrophil infiltration level was independently associated with clinical outcomes of advanced EGFR-mutant NSCLC received third-generation EGFR-TKI (Supplementary Fig. 17 and Supplementary Fig. 18).

## Discussion

The identification of activating EGFR mutations as actionable driver oncogenes revolutionized the management of advanced NSCLC, establishing a biomarker-driven treatment paradigm.^41^ Currently, third-generation EGFR-TKIs have become the standard of care for first-line treatment in patients with common EGFR mutations and second or above-line treatments to patients with acquired EGFR T790M mutation.^42^ Despite their marked efficacy, not all of them can benefit and resistance remains a critical fundamental challenge. Numerous studies have elucidated the underlying mechanisms of resistance including alterations in the target kinase, activation of signaling pathways through upregulation of downstream proteins and phenotypic transformation, etc.^43–47^ Nevertheless, nearly third of them showed unclear mechanisms of resistance. Considering the pivotal role of tumor immune microenvironment features and components in tumor proliferation, metastasis, and the development of drug resistance,^48–52^ it is valuable to investigate the relationships between tumor immune microenvironment features and components and efficacy of third-generation EGFR-TKIs in patients with EGFR-mutant NSCLC.

In the current study, we collected 50 paired pretreatment and post-resistant tumor tissue samples from 25 patients with EGFR T790M mutant NSCLC received third-generation EGFR-TKI from a phase II trial and 34 samples (22 pretreatment and 12 resistant) from 22 EGFR-mutant NSCLC patients received third-generation EGFR-TKIs in a real-world cohort. Multi-omics analysis were performed to dissect the tumor immune microenvironment features at baseline and after resistance to third-generation EGFR-TKIs treatment. Compared with pretreatment samples, we firstly observed that expression level of CXCR1 and CXCR2 were significantly increased in resistant samples, which were expressed by various immune and non-immune cells including neutrophils, macrophages, and endothelial cells.^53–57^ Then we analyzed the bulk RNA-seq and mIF data and found that resistant samples had a markedly enriched neutrophil infiltration. scRNA sequencing further identified a significant increase in CXCR1^+^ neutrophils, representing one terminus of neutrophil differentiation, in resistance samples. Compared to the CD52^+^ and S100A12^+^ neutrophils, CXCR1^+^ neutrophils exhibited unique characteristics, including higher PD-L1 expression and increased migration, chemotaxis, and activation. Additionally, we observed activation of pathways negatively regulating various immune cells (e.g., T cells, macrophages). Cell communication analysis demonstrated strong communication of CXCR1^+^ neutrophils with various cells, particularly enhanced communication with tumor cells and Tregs after drug resistance. Notably, we identified EGFR-related ligand-receptor pairs in CXCR1^+^ neutrophils and tumor cells, which were further enhanced in resistant samples, potentially contributing to EGFR-TKI resistance through sustained EGFR activation. Spatial analysis further showed that the average distance of CXCR1^+^ neutrophils to tumor cells markedly reduced from 33 to 19 μm and increased CXCR1^+^ neutrophils predominantly infiltrated into the tumor core in resistant samples. In line with this spatial architecture, Katey et al. found neutrophils distinguished a unique microenvironment associated with tumor progression.^58^ Collectively, these findings suggest that CXCR1^+^ neutrophils not only increase in number but also penetrate deeper into tumor core areas after resistance to third-generation EGFR-TKIs, thus promoting the establishment of an immunosuppressive tumor immune microenvironment.

Neutrophils, as the first-line defense against microbial infections, have the ability to orchestrate adaptive immune responses and chronic inflammation.^59,60^ Tumor-associated neutrophils play an implicated role in tumor angiogenesis, invasion, and metastasis through secretion of various growth factors, cytokines, and matrix-degrading enzymes.^61–67^ However, neutrophils could also initiate antitumor response by directly killing tumor cells or by interacting with other immunity components.^61,68,69^ The dual function of neutrophils within the tumor microenvironment suggests their heterogeneity and response plasticity to environmental cues. In our previous study, we observed that osimertinib could significantly increase neutrophil counts in the bronchoalveolar lavage fluids and lung tissues of EGFR-mutant lung cancer mouse model.^70^ Fang et al. reported that neurofilament heavy (NEFH) mutations, which were associated with reduced neutrophil infiltration, were enriched in TKI treatment-responsive samples.^34^ A more recent study showed that durable osimertinib response in EGFR mutant lung cancer required adaptive immunity with significantly decreased neutrophil content and increased intratumoral T cell content.^71^ These findings together emphasized the significance of neutrophils in response or resistance to EGFR-TKIs. In order to validate the role of CXCR1^+^ neutrophil mediating resistance to third-generation EGFR-TKIs and unravel the detailed mechansims, we performed in vitro and in vivo experiments using two well-known EGFR-mutant cell lines. The results showed that CXCR1^+^ neutrophils could significantly reduce osimertinib sensitivity. RNA-seq and Western blot analysis revealed that TNF-α/NF-κB signaling pathway and EMT-related markers’ expression were markedly up-regulated in resistant and co-cultured with CXCR1^+^ neutrophil groups. These effects could be neutralized by reparixin. These findings together suggest that CXCR1^+^ neutrophil could result in resistance to third-generation EGFR-TKIs via TNF-α/NF-κB signaling pathway (Fig. 8c).

Having noticed the significant role of CXCR1^+^ neutrophil infiltration in resistance to third-generation EGFR-TKIs, we then evaluated the predictive value of neutrophil infiltration abundance. We firstly divided the patients into long benefit and early disease progression groups using median PFS as cutoff. We found that patients from early disease progression group had a significant higher neutrophil infiltration than those in long benefit group. Then we stratified patients into high-risk and low-risk groups using the median CXCR1^+^ neutrophil infiltration level in pre-treatment samples as cutoff. The results showed that patients in the low-risk group had numerically higher ORR, dramatically longer PFS and OS than those in the high-risk group. These findings indicate that baseline neutrophil infiltration level was associated with the efficacy of third-generation EGFR-TKI in patients with EGFR-mutant NSCLC and could be considered as a potential predictive biomarker.

This study presents several limitations that should be noted. First, the small sample size and retrospective nature may introduce potential biases, including selection bias. Therefore, the results should be interpreted with caution, and further validation through large-scale prospective studies is warranted. Second, given the short lifespan of neutrophils, their proportion in scRNA sequencing is relatively low, posing a challenge for the comprehensive analysis of all neutrophil subpopulations. Third, while in vivo and in vitro experiments have shown that the combination of reparixin with third-generation EGFR-TKI effectively reverses drug resistance phenotype, this finding warrants careful consideration before being applied to clinical settings. Future investigations should delve deeper into the underlying mechanisms of CXCR1^+^ neutrophil infiltration and drug resistance, along with exploring potential therapeutic strategies to manipulate neutrophil infiltration and its associated molecular mechanisms.

In summary, the current findings suggest that CXCR1^+^ neutrophil infiltration in pretreatment tumor tissues was associated with the efficacy of third-generation EGFR-TKI and increased CXCR1^+^ neutrophil infiltration in posttreatment tumor tissues played a role in mediating the resistance to third-generation EGFR-TKI in EGFR-mutant NSCLC patients, underscoring the clinical significance of CXCR1^+^ neutrophils within the tumor microenvironment. Our findings may aid in identifying patients more likely to benefit from third-generation EGFR-TKI treatment.

## Materials and methods

### Ethics approval and consent to participate

The study protocol received approval from our center’s ethics committee and institutional review board, and was conducted in compliance with the Declaration of Helsinki, Guidelines for Good Clinical Practice, and relevant Chinese laws and regulations.

### Patients’ inclusion

This study incorporated 25 NSCLC patients harboring T790M mutations (Fig. 1a). All of them received SH-1028 treatment at our center from April 2019, to February 2022. The study included participants who met the following key eligibility criteria: (i) have a confirmed diagnosis of locally advanced or metastatic NSCLC through histological or cytological analysis, (ii) be 18 years or older, (iii) possess at least one measurable lesion as defined by Response Evaluation Criteria in Solid Tumors, version 1.1 (RECIST v.1.1), (iv) hold an Eastern Cooperative Oncology Group (ECOG) performance status score between 0 and 2, (v) show disease progression after receiving first- or second-generation EGFR-TKI therapy, and (vi) provide written informed consent. Additionally, patient involvement required confirmation of either an acquired or primary EGFR T790M mutation in tumor tissue or pleural effusion cells. Patients were deemed ineligible if they fulfilled any of the subsequent criteria: (i) previous exposure to third-generation EGFR-TKIs; (ii) administration of cytotoxic chemotherapy or other anticancer agents within a 14-day window preceding the initial dose of SH-1028; (iii) receipt of investigational drugs within 14 days or 5 half-lives prior to the commencement of SH-1028 treatment; (iv) inadequate bone marrow and organ function; (v) established medical history of hypersensitivity reactions towards any constituent of SH-1028.

### Data collection

Data of eligible participants were collected from electronic medical records, adhering to the standard requirements for patient follow-up data during treatment, including treatment responses and clinical outcomes. Baseline characteristics included age, gender, smoking status, lung cancer histology based on WHO classification, and EGFR alteration status. EGFR alterations were identified by the ADx-ARMS EGFR Detection Method (Amoy Diagnostics Co., Ltd., Xiamen, China). Tumor response was evaluated one month post-therapy initiation and subsequently every two months, in alignment with the RECIST v.1.1. The assessment of treatment response included complete response (CR), partial response (PR), stable disease (SD), or progressive disease (PD). The cut-off date for the last follow-up was February 13, 2023.

### Transcriptomic analysis

Tumor biopsy tissues were procured at pretreatment and upon manifestation of drug resistance, resulting in a total of 50 samples that underwent whole transcriptomic sequencing (Fig. 1). RNA was isolated from formalin-fixed, paraffin-embedded (FFPE) tissues using a RNeasy kit (Qiagen, USA), and its quality was confirmed using a NanoDrop 2000 (Thermo Scientific, USA) and an Agilent 2100 Bioanalyzer. This RNA served to build cDNA libraries, which then underwent RNA sequencing. Post-quality assessment, 36 samples met the criteria (17 from the pretreatment and 19 resistance samples) for further analysis. The CIBERSORT algorithm was utilized to estimate the relative proportions of 22 immune cell types within the tumor microenvironment using gene expression data. The X-cell algorithm was applied to validate the results of CIBERSORT algorithm. We identified differentially expressed genes (DEGs) between pretreatment and drug-resistant tumor samples using the DESeq2 algorithm, with an adjusted *P*-value < 0.05 and |log2(Fold Change)| > 1.2 deemed statistically significant. For each gene, the expression score was equated to the gene’s expression level. DEGs were subsequently subjected to Gene Ontology (GO) enrichment and Kyoto Encyclopedia of Genes and Genomes (KEGG) pathway analyses using clusterProfiler.^72^ We carried out Gene set enrichment analysis (GSEA) on all pre-ranked genes. GO terms, KEGG pathways and GSEA enrichment scores with false discovery rates (FDRs) < 0.05 were considered significantly enriched. We performed protein-protein interactions using STRING (https://string-db.org/) and Cytoscape software.

### Multiplexed immunofluorescence

The expression level of CD66b, PD-L1, CD8, PD-1, CXCR1, and PanCK in paired pretreatment and posttreatment tumor tissues was assessed using multiplexed immunofluorescence (mIF). Staining procedures were executed utilizing the BOND RX fully automated stainers (Leica Biosystems, Wetzlar, Germany), and image acquisition was facilitated through the Vectra Polaris system (PerkinElmer, Waltham, MA). A range of primary antibodies was employed, including anti-CD66b (ab197678, Abcam), anti-PD-L1 (13684S, Cell Signaling Technology), anti-CD8 (ab237709, Abcam), anti-PD-1 (A20217, ABclonal), anti-CXCR1 (ab124344, Abcam), and anti-PanCK (ab234297, Abcam). Following each staining cycle, heat-induced epitope retrieval was performed to eliminate both primary and secondary antibodies. Areas positive for PanCK were designated as tumor regions, whereas PanCK-negative areas were identified as stromal regions. The selection of representative regions of interest was conducted by an experienced pathologist. Multiple fields of view at a 20× resolution were captured as multispectral images. Cell identification within these images was achieved through supervised machine learning algorithms in Qupath software. Both the thresholds for staining and the accuracy of phenotypic algorithms were meticulously optimized and validated by two experienced pathologists for each individual case.

### Flow cytometry

Neutrophils were isolated from fresh blood using CD66b microbeads (130-104-913, MACS). The isolated cells were then stained with corresponding antibodies at the recommended concentrations by the manufacturers, at 4 °C for 45 min, followed by washing with PBS. Staining was conducted using CD66b-APC (ab275586, Abcam) and CXCR1-Alexa Fluor 700 (FAB330N, R&D Systems) to label the target cells. For mouse models, neutrophils were isolated from tumors using Ly6g microbeads (130-120-337, Miltenyi). The isolated cells were then stained with corresponding antibodies at the recommended concentrations by the manufacturers, at 4 °C for 45 min, followed by washing with PBS. Staining was conducted using Ly6g-Alexa Fluor 647 (127610, Biolegend) and CXCR1-PE (566383, BD Pharmingen) to label the target cells.

### Single-cell RNA sequencing

Single-cell RNA sequencing was performed on 34 samples (22 pretreatment and 12 resistant) from 22 EGFR mutant NSCLC patients received third-generation EGFR-TKIs in a real-world cohort. The protocol for this study received approval from the Ethics Committee and Institutional Review Board of Shanghai Pulmonary Hospital (No. K18-136). Each sample underwent mincing and was transferred to DMEM (ThermoFisher) supplemented with 0.5% collagenase I/IV (Roche), 20U/μl DNAse I (Sigma), and 1 mg/ml neutral protease (Invitrogen). Samples were then incubated at 37 °C for 15–30 min with manual shaking every 5 minutes. Subsequently, ice-cold PBS (ThermoFisher) was added, and samples were filtered using Scienceware Flowmi 70-μm cell strainers (VWR). After centrifugation (300 g, 4 °C, 5 min), the cell pellet was resuspended in red blood cell lysis buffer (Sigma) for 2 min at 25 °C, followed by another centrifugation step (300 g, 4 °C, 5 min). Cells were then resuspended in PBS containing 400 μg UltraPure BSA (ThermoFisher), filtered over Scienceware Flowmi 40-μm cell strainers (VWR), and counted using a TC20TM Automated Cell Counter (Bio-Rad). Samples were processed within 3 hours from surgical removal and profiled using the Chromium Single Cell 3’ Library & Gel Bead Kit v3 (10X Genomics). Raw gene expression matrices were generated for each sample using CellRanger 3.1.0, then mapped in R 4.3.1 with GRCh38 as reference, and converted to a Seurat object using the Seurat (V4.0).^73^ Mitochondrial gene percentage was evaluated using the PercentageFeatureSet(object, pattern = “^MT-“) function, and those cells with a mitochondrial gene percentage over 20% were excluded. After excluding genes in a blacklist,^74^ a list of the top 3000 genes with highly variable genes (HVG) was compiled using the FindVariableFeatures. Harmony was performed to integrate all cells (V0.1.0).^75^ Clustering analysis was then performed, and groups were annotated using marker genes.

### Trajectory inference of cell states

The cell lineage trajectory of neutrophils was inferred using Monocle2 (V2.14) with marker genes having a q value < 0.001 calculated by the differentialGeneTest function.^76^ Monocle was used to infer the differentiation trajectory after cell ordering.

### Single-cell metabolism quantification

In order to identify the prominent metabolic pathway among neutrophils, we quantified the major metabolism subtypes using the scMetabolism program.^77^ Default values were set for all other parameters.

### Cell‑cell interaction analysis

We utilized Cellchat^78^ to infer cellcell interaction between various cell types. The approach estimates the potential interaction signal among these cell subsets by analyzing gene expression and determines significance using permutation tests. Based on a permutation test, significant ligand-receptor pairs with P value 0.01 were extracted from two cell subsets.

### Cell culture

The H1975 and PC-9 cell lines were obtained from the Shanghai Academy of Science (Shanghai, China). H1975_OR and PC_9OR cells were constructed in our laboratory.These cells were cultured with RPMI 1640 medium (Gibco, United States). In the complete medium (Gibco, United States), 10% fetal bovine serum was used, along with 1% penicillin-streptomycin. Neutrophils were harvested from tumor sample in BALB/C-Nude mice by negative selected magnetic beads (70907, BEAVER). CXCR1-PE (566383, BD Pharmingen) was initially conjugated with PE-tagged magnetic beads (130-048-801, Miltenyi) and incubated, following which the complex is utilized to perform sorting of neutrophils, thereby facilitating the separation of CXCR1^+^ and CXCR1^-^ neutrophil populations. All cells were maintained at 37 °C in an environment containing 5% carbon dioxide.

### ELISA

The neutrophils culture medium was collected and centrifuged at 1000 × g for 10 min at 4 °C. The cells were then removed to obtain the supernatant. Adding 300 μL of 1x wash buffer to each well, let it sit for 40 s, then discarded the liquid. We repeated this wash three times. Adding 100 μL of standard/sample dilution buffer to blank wells, and 100 μL of standards or samples to the other wells. The plate was sealed and incubated at 37 °C for two hours. Biotinylated antibody working solution (100×) was prepared 15 min before use. After incubation, to discard the liquid, wash the wells, add 100 μL of the antibody solution, seal, and incubate at 37 °C for one hour. We prepared the streptavidin-HRP (100×) 15 minutes before use. Discarded the liquid, washed again, added 100 μL of streptavidin-HRP, sealed and incubated at 37 °C for 30 min. Pre-warm the microplate reader. Discarded the liquid, washed and added 100 μL of TMB substrate, incubating at 37 °C for 15–20 min in the dark. A volume of 50 μL of stop solution was added and measured within five minutes.

### Immunoblotting

Using RIPA buffer (Epizyme) containing protease and phosphatase inhibitor cocktails, cell samples were lysed. Assaying protein concentrations with bicinchoninic acid (BCA) protein assay kits (Epizyme) was performed. The cell lysate (15–30 g) was applied to polyacrylamide gels with 0.1% sodium dodecyl sulfate (SDS) and then transferred to polyvinylidene fluoride (PVDF) membranes(Epizyme). After blocking with block solution (Epizyme) at room temperature for 30 min, membranes were incubated overnight at 4 °C with antibodies diluted in commercial antibody diluent (Epizyme). The following primary antibodies were used. GAPDH Rabbit mAb (A19056, ABclonal), Vimentin Rabbit mAb (A19607, ABclonal), E-Cadherin Rabbit mAb (A22333, ABclonal), NF-kB p65/RelA Rabbit mAb (A19653, ABclonal), Phospho-NF-κB p65/RelA-S536 Rabbit mAb (AP1294, ABclonal).

### Cell viability assay

Cells were plated in triplicate into 96-well plates the day prior and subsequently treated with varying concentrations of osimertinib for an additional 72 h. Following the treatment period, cells were stained using Cell Counting Kit-8 (CCK-8, Beyotime) reagent and analyzed using a Varioskan Flash (450 nm, Thermo) after a one-hour incubation. After subtracting the blank control fluorescence, we normalized the fluorescence signal to derive the growth ratio.

### Animal models

Five-week-old male nude mice were obtained from SLAC LABORATORY (Shanghai, China) for use in the study. These mice received a subcutaneous injection of 5 × 10^6^ cells and monitored until tumors became visible. In the H1975_OR and PC-9_OR xenograft models, once tumor volumes reached 200 mm^3^, mice (*n* = 4 per group) were treated according to the experimental design with PBS, osimertinib (2.5 mg/kg body weight, administered daily via oral gavage), and/or reparixin (25 mg/kg body weight, given intraperitoneally every two days).

### Intravital imaging of tumors using DAOSLIMIT

To investigate CXCR1^+^ neutrophils in live mice, DAOSLIMIT, an optical instrument developed at Tsinghua University (Beijing, China), was utilized for intravital imaging over hour-long sessions.^79^ Mice were prepared according to the medical modeling protocols outlined in the figures and then subjected to DAOSLIMIT imaging. WGA (5 μg, W11261, Thermo Fisher), Ly6G (3 μg, 127610, BioLegend), CXCR1 (3 μg, 566383, BD Pharmingen), and phosphate-buffered saline (PBS, 60 μL) were injected into the mice via tumor vasculature. Throughout the DAOSLIMIT imaging process, a 37 °C body temperature control device was used to maintain the mice’s physiological conditions. For the data collection, sLFdriver software was employed to capture DAOSLIMIT data from randomly selected tumor regions, with subsequent analysis and visualization performed using our customized MATLAB tool.

## Statistical analysis

The clinicopathologic characteristics were summarized numerically and by percentages. Categorical variables were compared using the Chi-square test or the Fisher’s exact test when necessary. The Mann-Whitney U test analyzed continuous variables. PFS was characterized as the time span from the commencement of SH-1028 treatment until the onset of systemic progression (inclusive of intracranial progression) or death, whichever occurred first, with the measurement censored at the date of the last tumor evaluation. OS was determined as the duration from the initiation of SH-1028 treatment to either the date of death from any cause or to the last follow-up date, whichever was earlier. The analysis of outcome disparities was conducted using Kaplan-Meier curves, supplemented by two-sided log-rank tests for statistical assessment. All statistical analyses were conducted using R (v4.2.3) and Qupath (v5.0). A two-sided *P*-value < 0.05 was considered statistically significant.

## Supplementary information

Supplementary Materials

Movie S1

Movie S2

## Data Availability

The raw RNA-seq and single-cell RNA sequencing data reported in this paper have been deposited in the Genome Sequence Archive (Genomics, Proteomics & Bioinformatics 2021) in National Genomics Data Center, Chinese Academy of Sciences (GSA: HRA009004; GSA: HRA009043) that are publicly accessible at https://ngdc.cncb.ac.cn/gsa-human. Any other data employed in this study, both utilized and analyzed, are available upon a reasonable request to the corresponding author.
