# Supplementary material for: CXCR1+ neutrophil infiltration orchestrates response to third-generation EGFR-TKI in EGFR mutant non-small-cell lung cancer
Source: Signal Transduct Target Ther. 2024 Dec 6;9:342. doi: 10.1038/s41392-024-02045-2 (PMC11621634; doi:10.1038/s41392-024-02045-2)
Supplement: Supplementary file 1 — Supplementary Materials [file 41392_2024_2045_MOESM1_ESM.pdf]

Supplementary Materials for

CXCR1 + neutrophil infiltration orchestrates response to third-generation EGFR-TKI in EGFR mutant NSCLC

Haowei Wang, Anwen Xiong, Xiaoxia Chen, Junhong Guo, Zhuoran Tang, Chunyan Wu, Shengxiang Ren, Caicun Zhou, Jian Chen, Likun Hou, Tao Jiang
Correspondence to: tonyjiangdr@163.com

This PDF file includes:

Figures. S1 to S18
Tables S1 to S2
Captions for Movies S1 to S2

Other Supplementary Materials for this manuscript include the following:

Movies S1 to S2

a

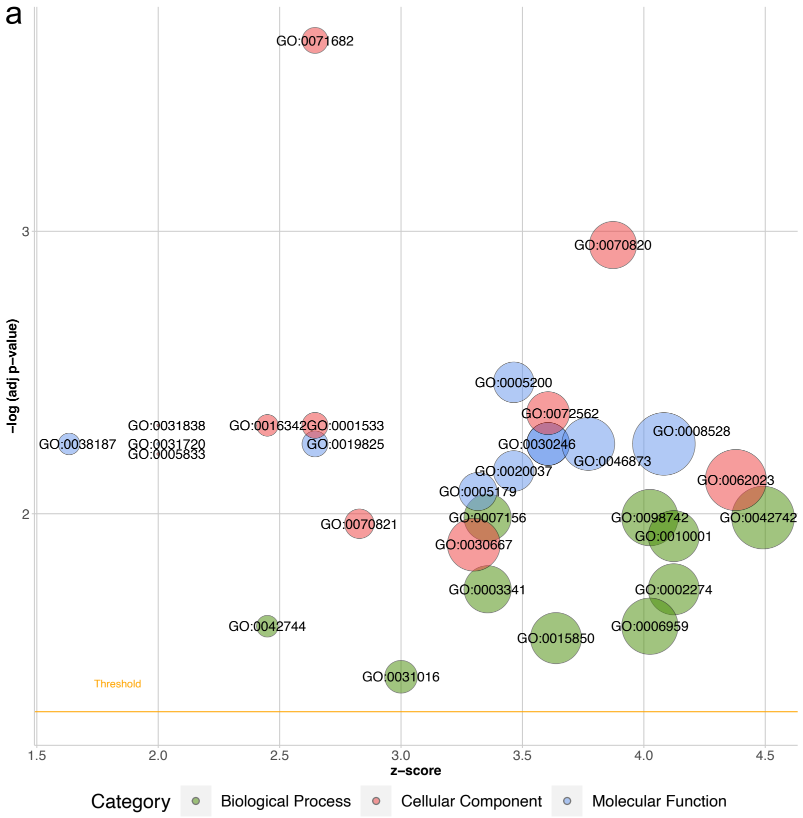

ID	Description
GO:0042742	defense response to bacterium
GO:0098742	cell−cell adhesion via plasma−membrane adhesion molecules
GO:0007156	homophilic cell adhesion via plasma membrane adhesion molecules
GO:0010001	glial cell differentiation
GO:0002274	myeloid leukocyte activation
GO:0003341	cilium movement
GO:0006959	humoral immune response
GO:0042744	hydrogen peroxide catabolic process
GO:0015850	organic hydroxy compound transport
GO:0031016	pancreas development
GO:0005200	structural constituent of cytoskeleton
GO:0038187	pattern recognition receptor activity
GO:0019825	oxygen binding
GO:0008528	G protein−coupled peptide receptor activity
GO:0031720	haptoglobin binding
GO:0001653	peptide receptor activity
GO:0030246	carbohydrate binding
GO:0046873	metal ion transmembrane transporter activity
GO:0020037	heme binding
GO:0005179	hormone activity
GO:0071682	endocytic vesicle lumen
GO:0070820	tertiary granule
GO:0072562	blood microparticle
GO:0016342	catenin complex
GO:0001533	cornified envelope
GO:0031838	haptoglobin−hemoglobin complex
GO:0005833	hemoglobin complex
GO:0062023	collagen−containing extracellular matrix
GO:0070821	tertiary granule membrane
GO:0030667	secretory granule membrane

b

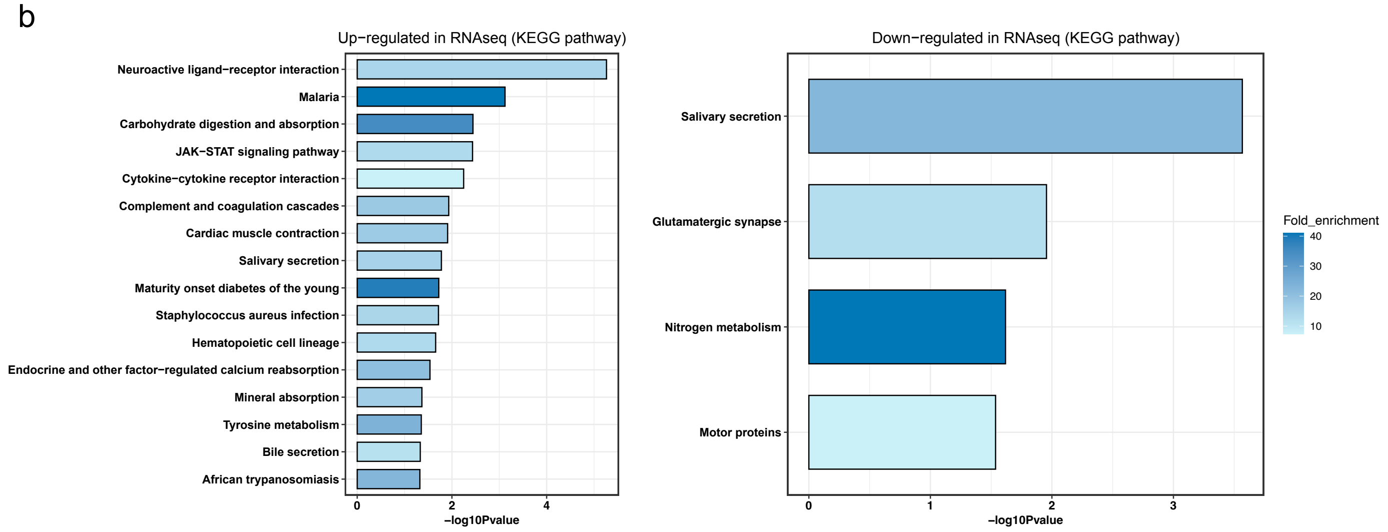

Figure. S1.

GO (**a**) and KEGG (**b**) enrichment analysis of different expression genes between pretreatment and resistant samples.

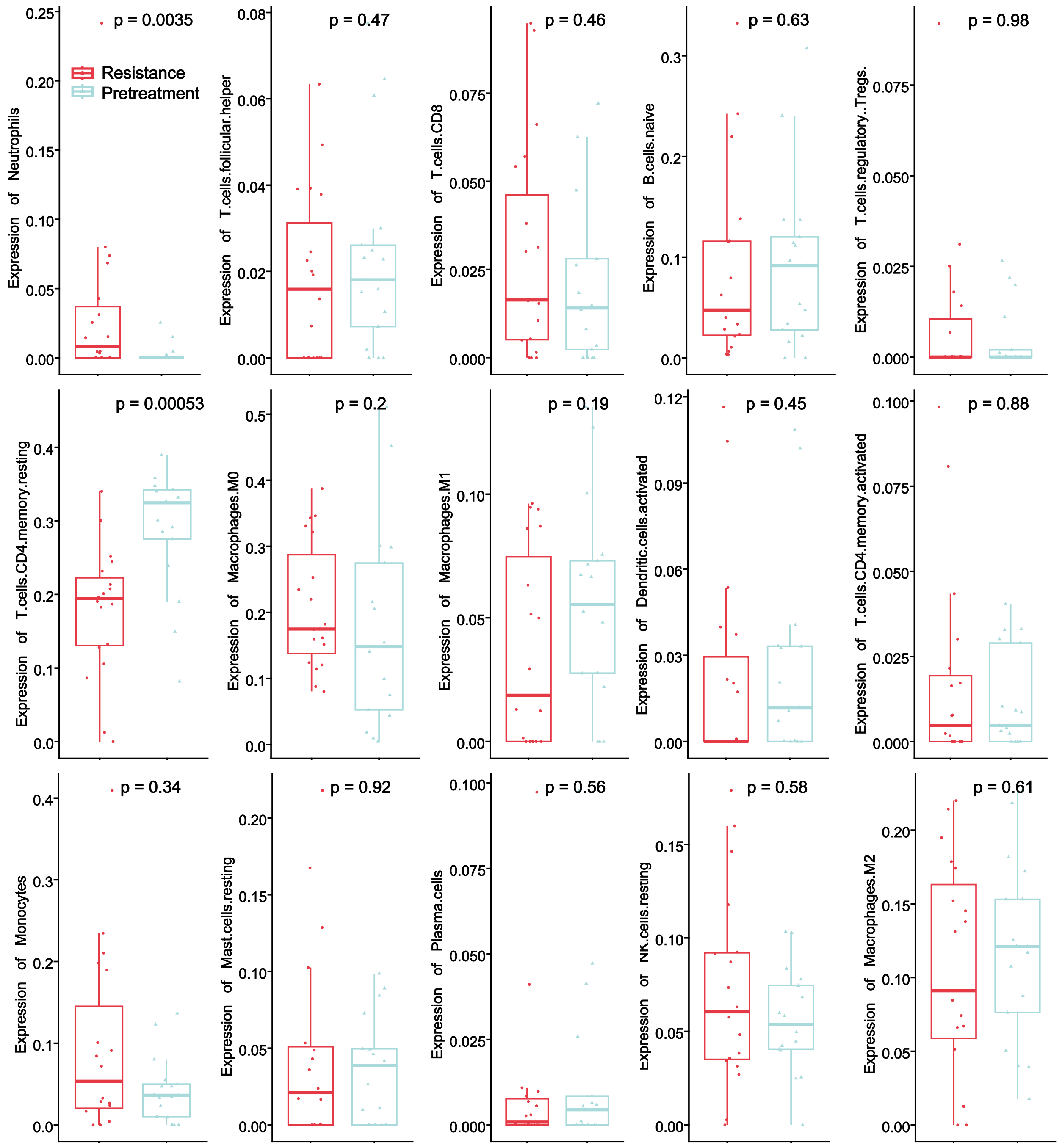

Figure. S2.

Comparison of each immune cell infiltration abundance between pretreatment and resistant samples.

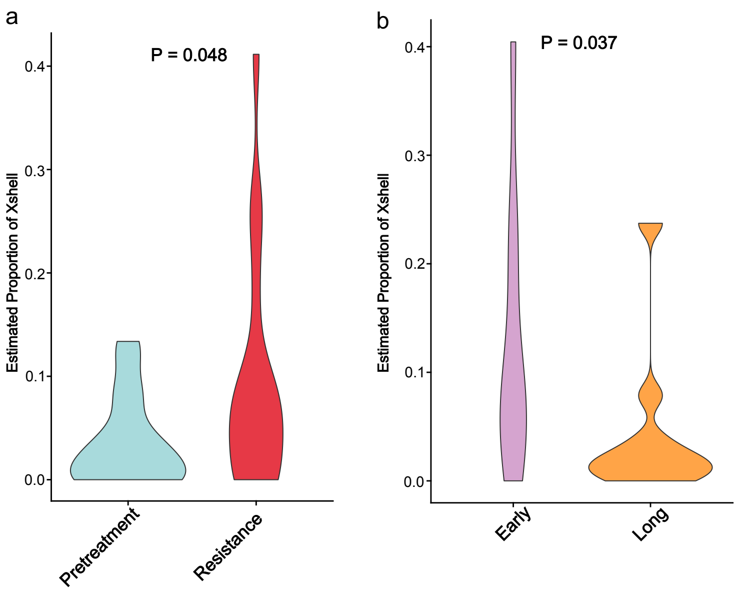

Figure. S3.

Comparison of the infiltration levels of neutrophils between pretreatment and resistant groups (**a**), and early progression and long benefit groups (**b**) by Xcell.

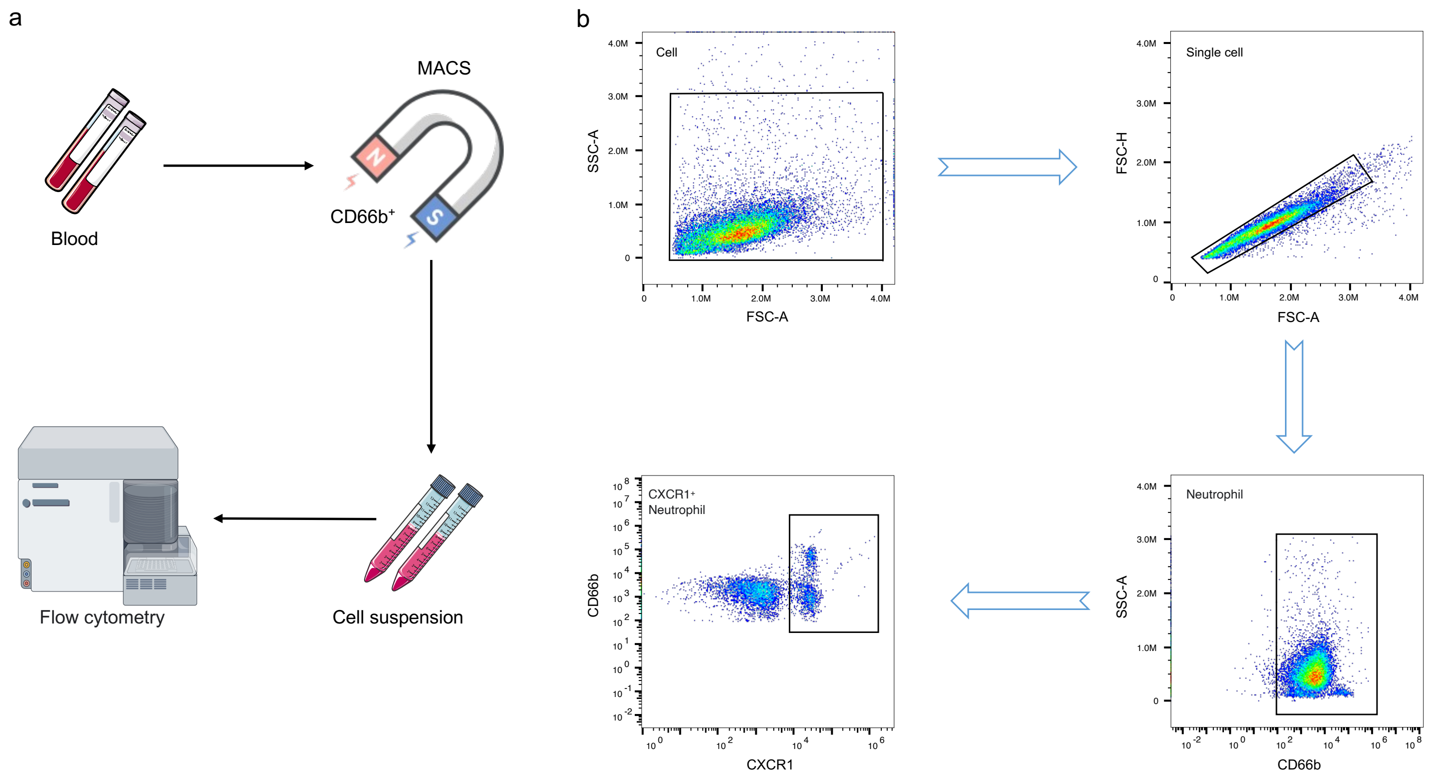

Figure. S4.

(**a**) Neutrophils were sorted with CD66b microbeads (the graphical model figure was created using Figdraw). (**b**) Flow cytometry gating strategy diagram.

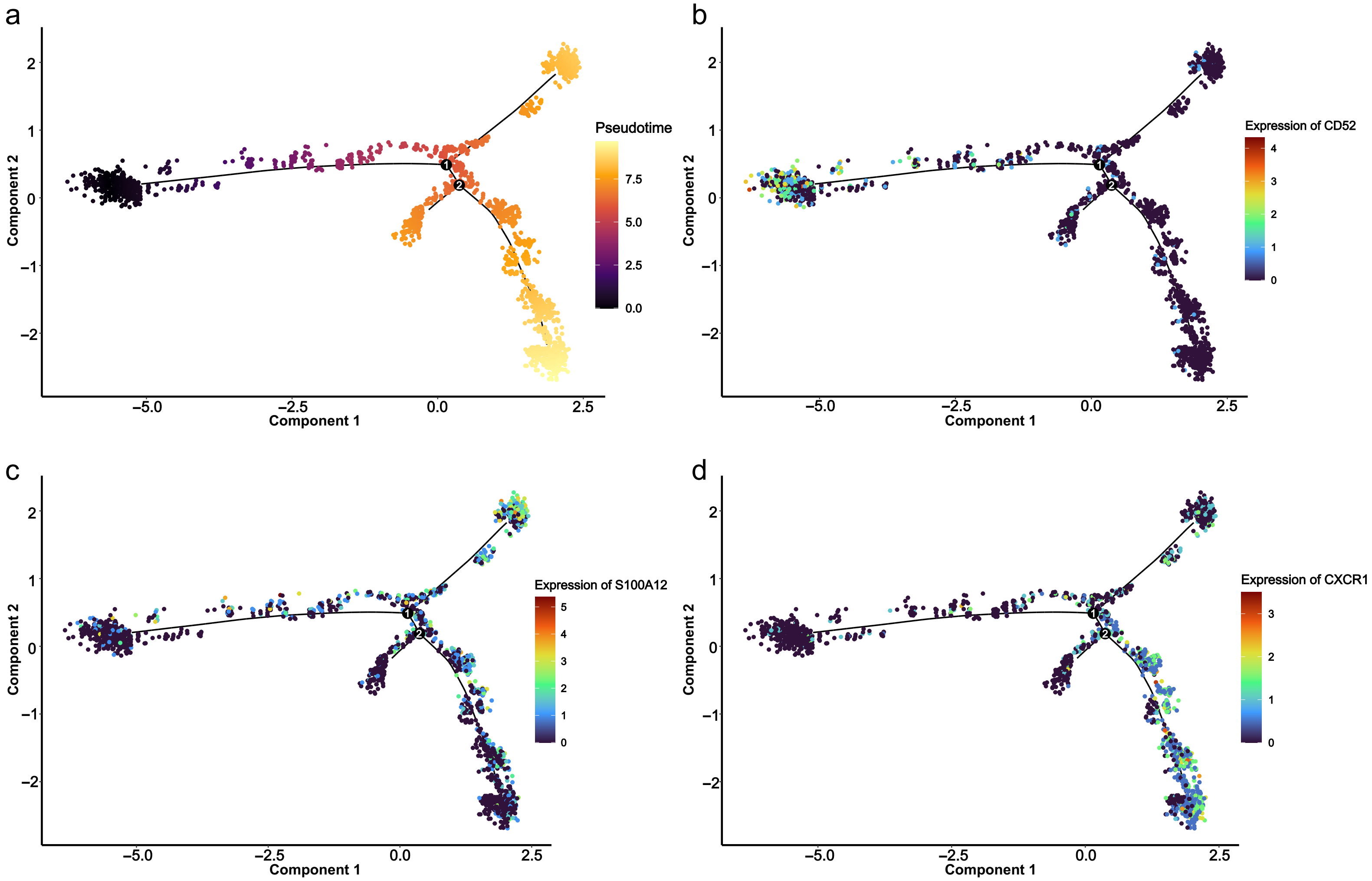

Figure. S5.

(**a**) The pseudotime value according neutrophil trajectories detected by Monocle2. The gene expression value of CD52 (**b**), S100A12 (**c**) and CXCR1 (**d**) mapped in the trajectory.

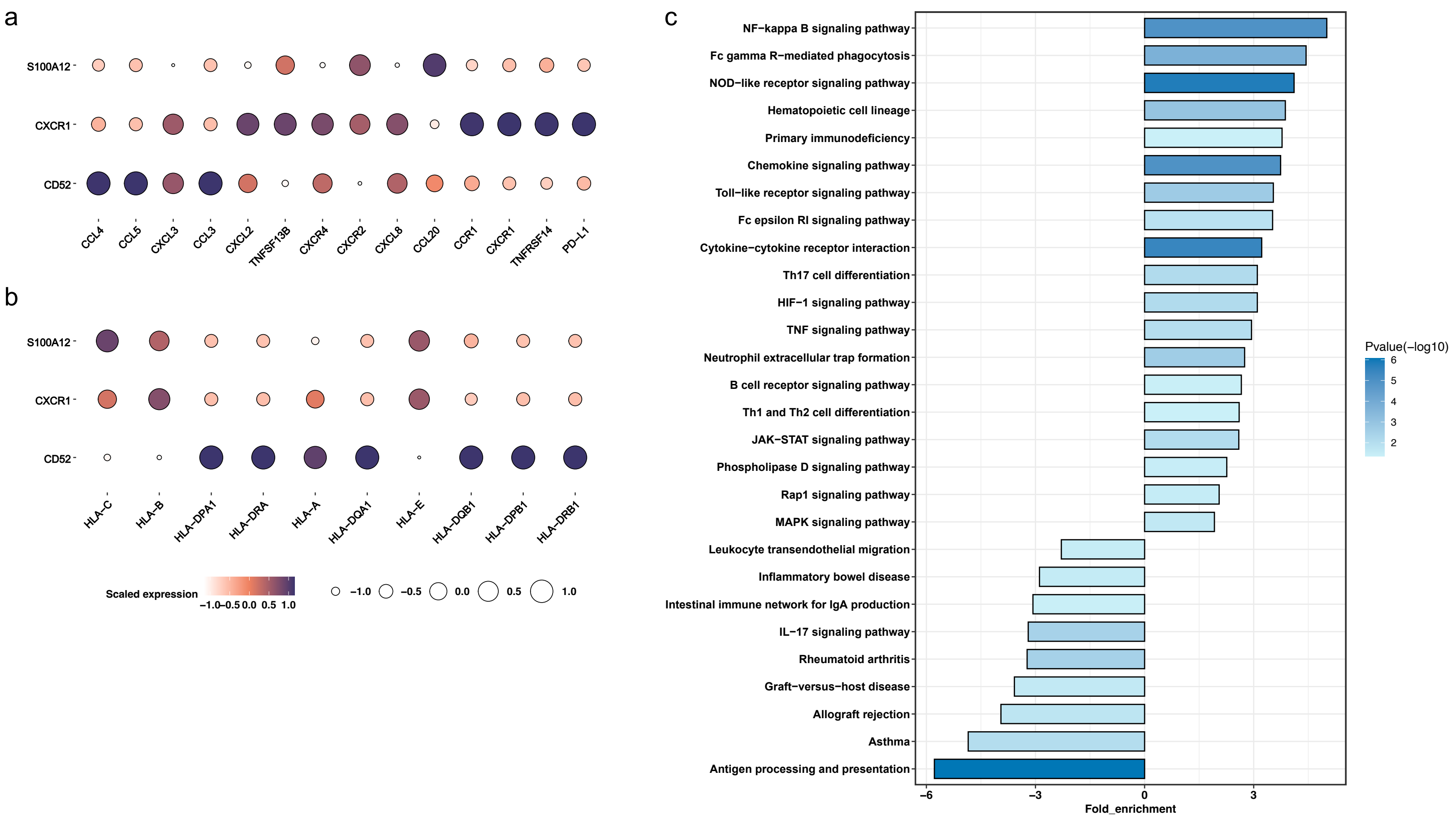

Figure. S6.

(**a**) Expression profiles of differentially expressed cytokines. (**b**) Expression profiles of differentially expressed MHC molecules. (**c**) KEGG enrichment analysis in CXCR1^{+} neutrophils.

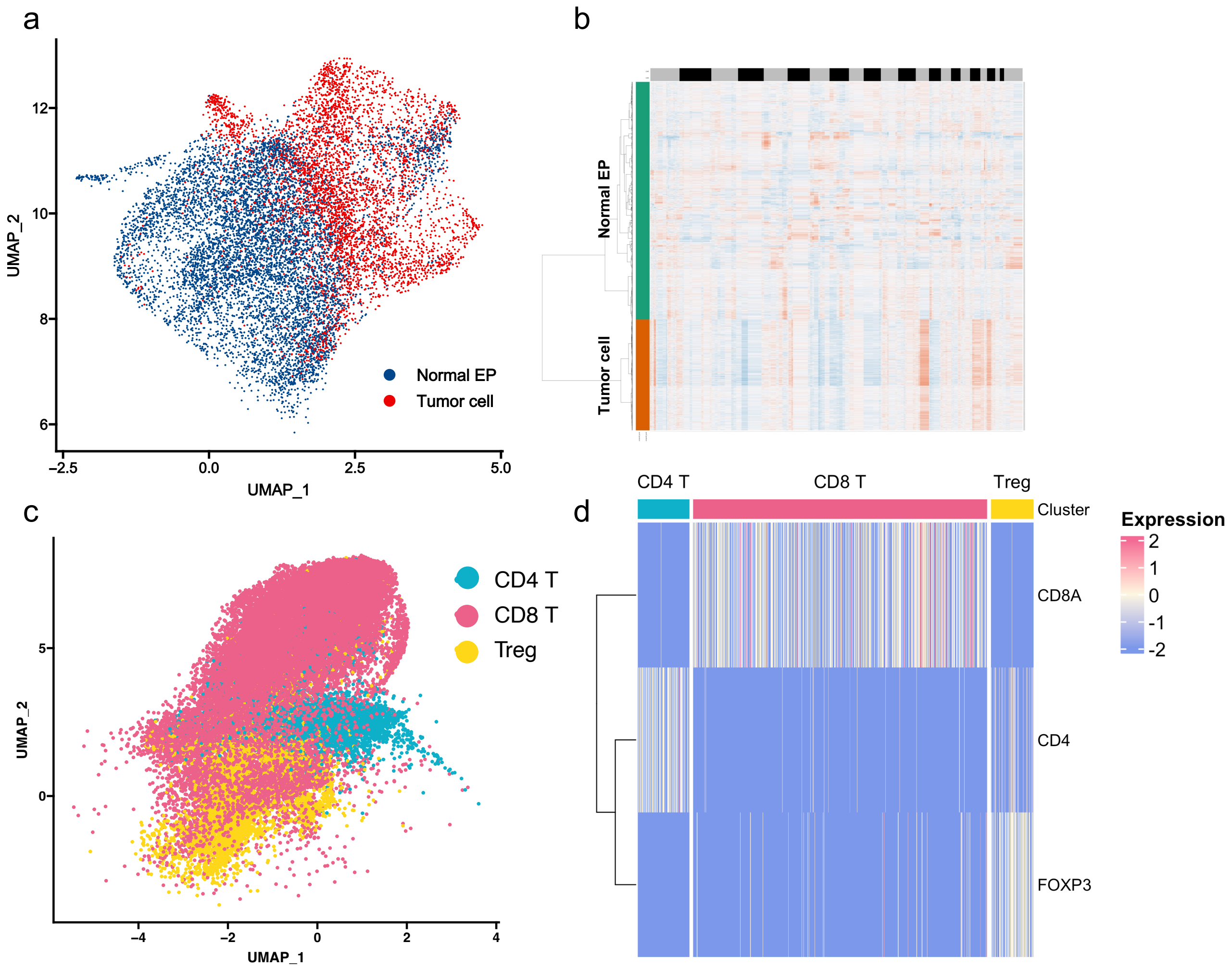

Figure. S7.

(**a**) The UMAP plot of normal epithelial cell and tumor cell. (**b**) Copykat algorithm was performed to distinguish normal epithelial cell and tumor cell. (**c**) The UMAP plot of CD8 T cell, CD4 T cell and Treg. (**d**) Heatmap of marker genes.

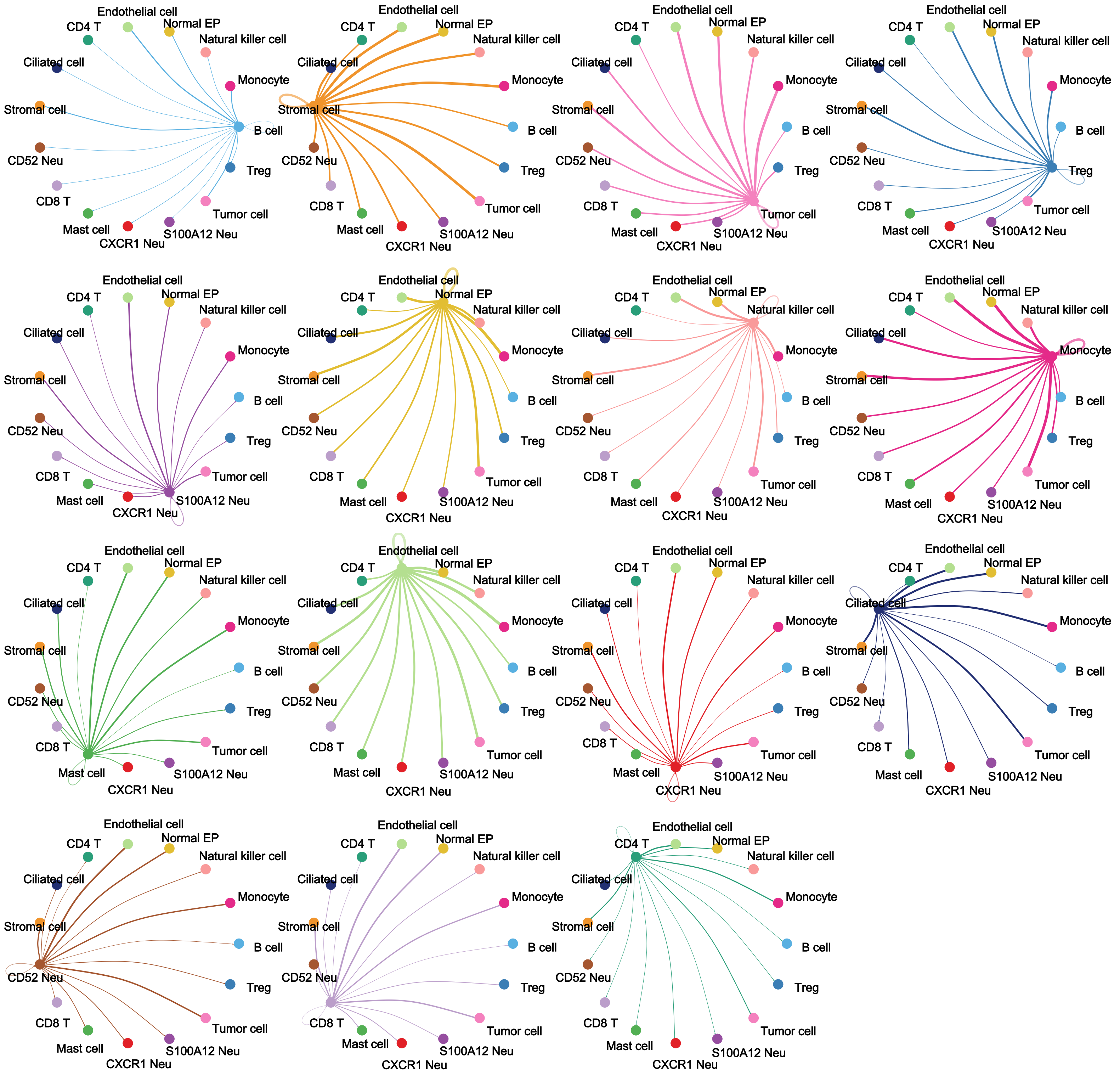

Figure. S8.

Communication patterns among different cell types.

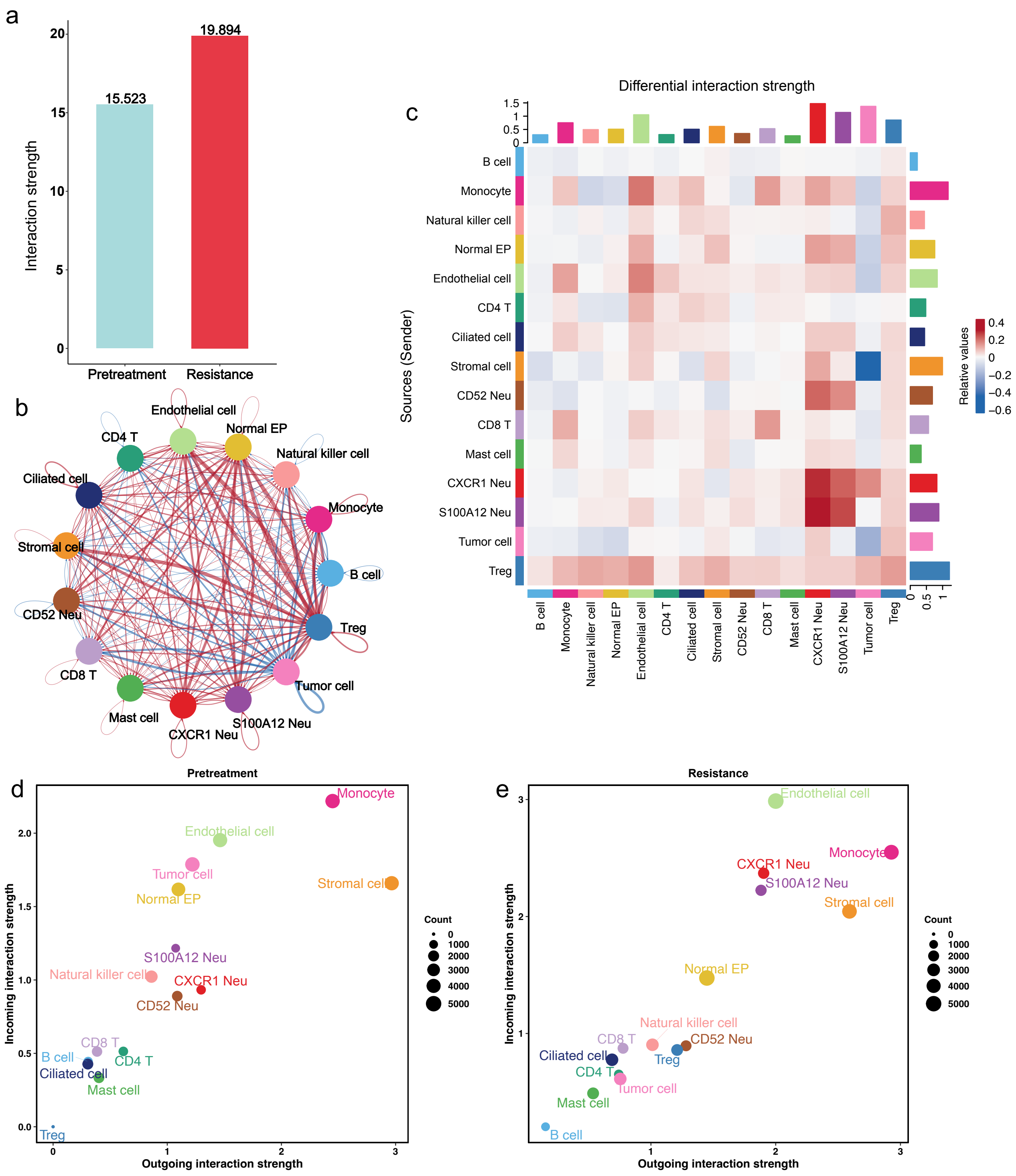

Figure. S9.

(**a**) Cell interaction strength in pretreatment and resistance. The comparison of interaction strength between pretreatment and resistance among various cells (Red: upregulation; Blue: downregulation) in circle chart (**b**) and heatmap (**c**). Dotplot of outgoing and incoming interaction strength in pretreatment (**d**) and resistance (**e**).

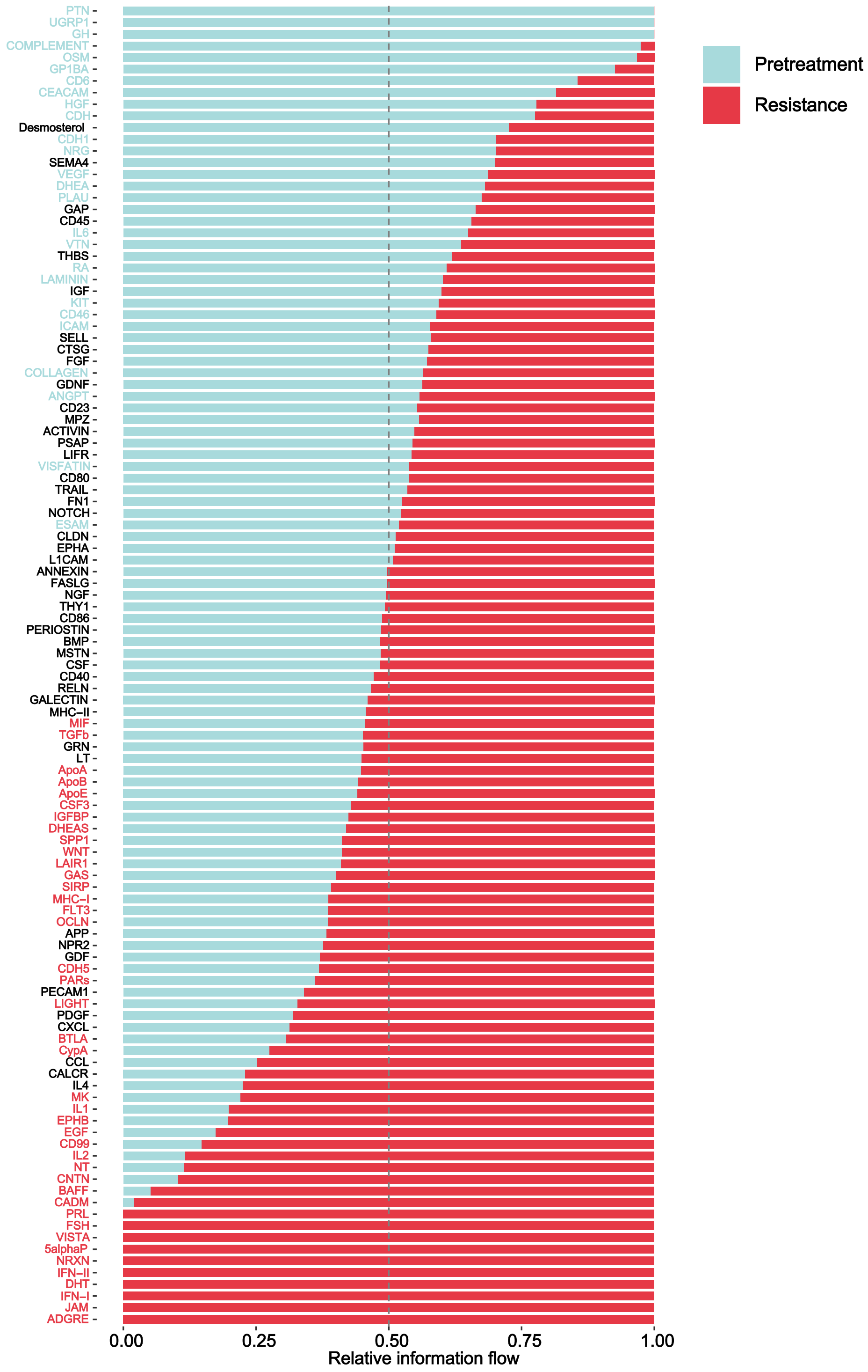

Figure. S10.

The enriched pathways in pretreatment and resistance in CellChat.

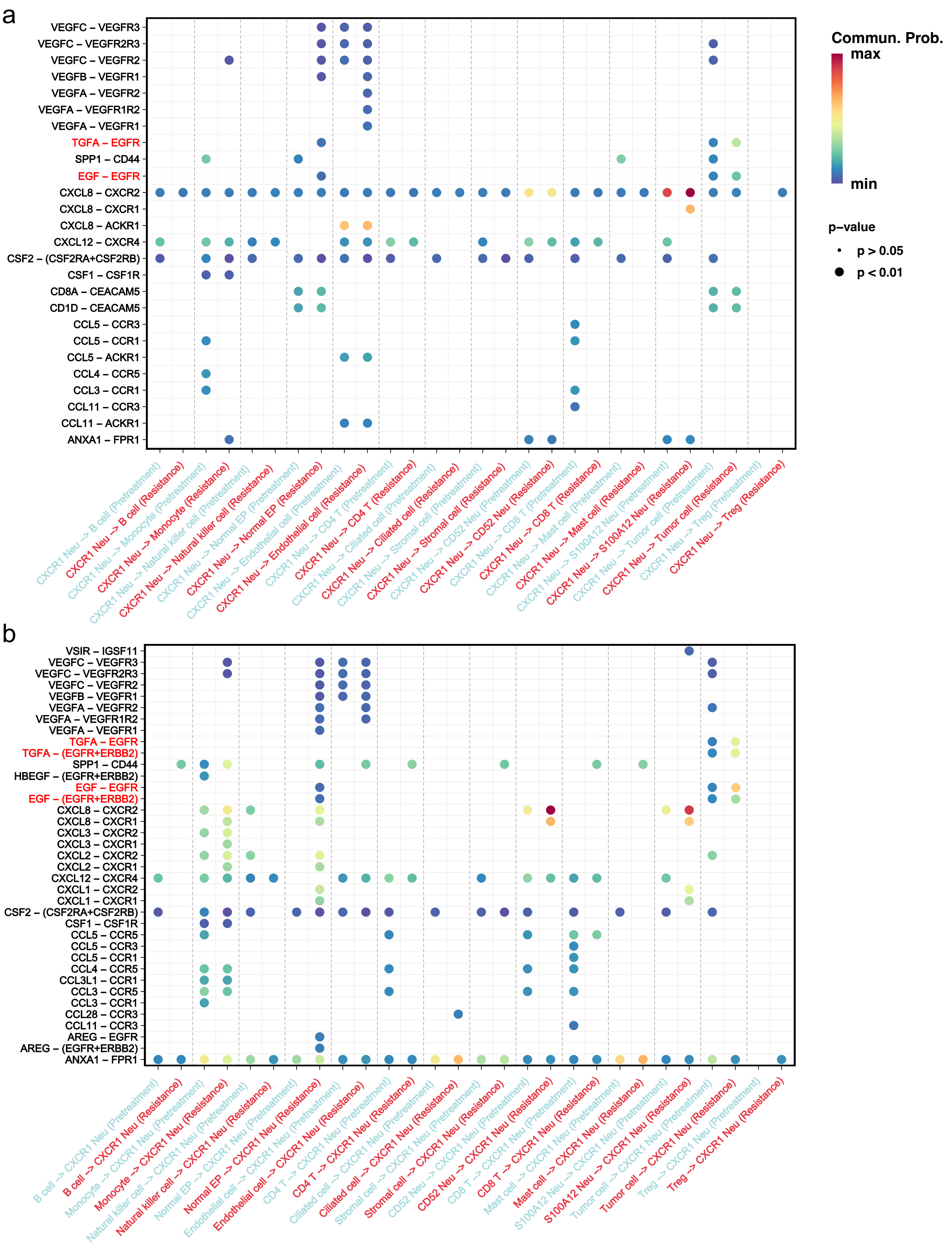

Figure. S11.

The comparison of significant ligand-receptor pairs in $CXCR1^{+}$ neutrophil as outputs (**a**) and inputs (**b**).

a

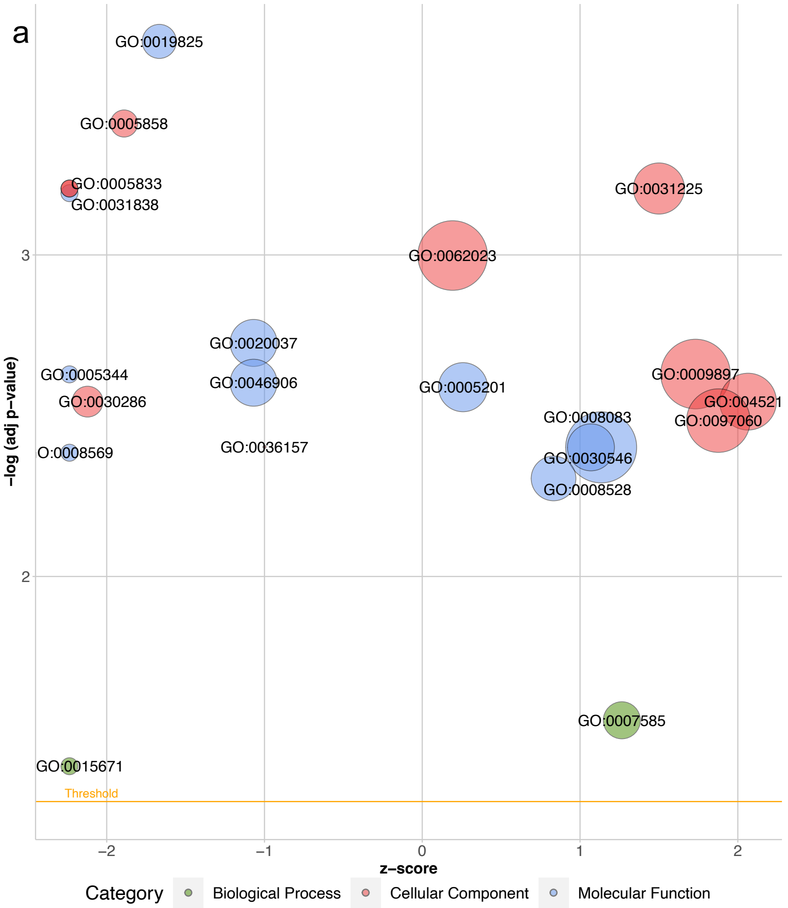

ID	Description
GO:0007585	respiratory gaseous exchange by respiratory system
GO:0015671	oxygen transport
GO:0019825	oxygen binding
GO:0031720	haptoglobin binding
GO:0020037	heme binding
GO:0005344	oxygen carrier activity
GO:0046906	tetrapyrrole binding
GO:0005201	extracellular matrix structural constituent
GO:0030546	signaling receptor activator activity
GO:0008083	growth factor activity
GO:0008569	minus−end−directed microtubule motor activity
GO:0008528	G protein−coupled peptide receptor activity
GO:0005858	axonemal dynein complex
GO:0031838	haptoglobin−hemoglobin complex
GO:0031225	anchored component of membrane
GO:0005833	hemoglobin complex
GO:0062023	collagen−containing extracellular matrix
GO:0009897	external side of plasma membrane
GO:0030286	dynein complex
GO:0045211	postsynaptic membrane
GO:0097060	synaptic membrane
GO:0036157	outer dynein arm

b

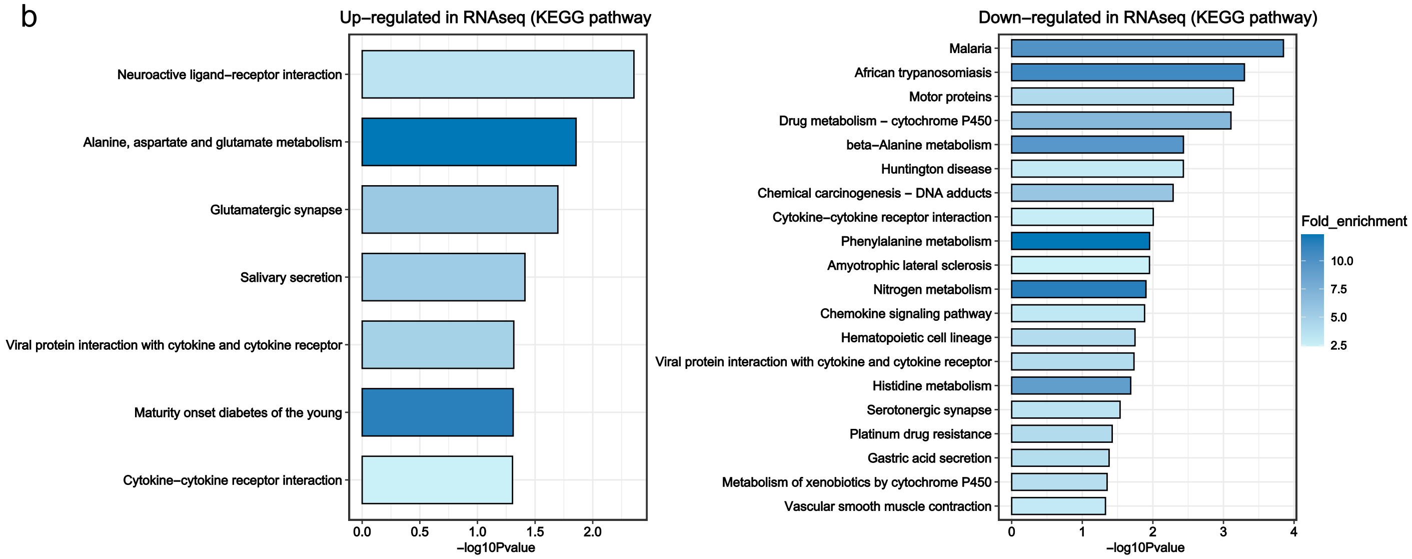

Figure. S12.

GO (**a**) and KEGG (**b**) enrichment analysis of different expression genes between long benefit and early disease progression groups.

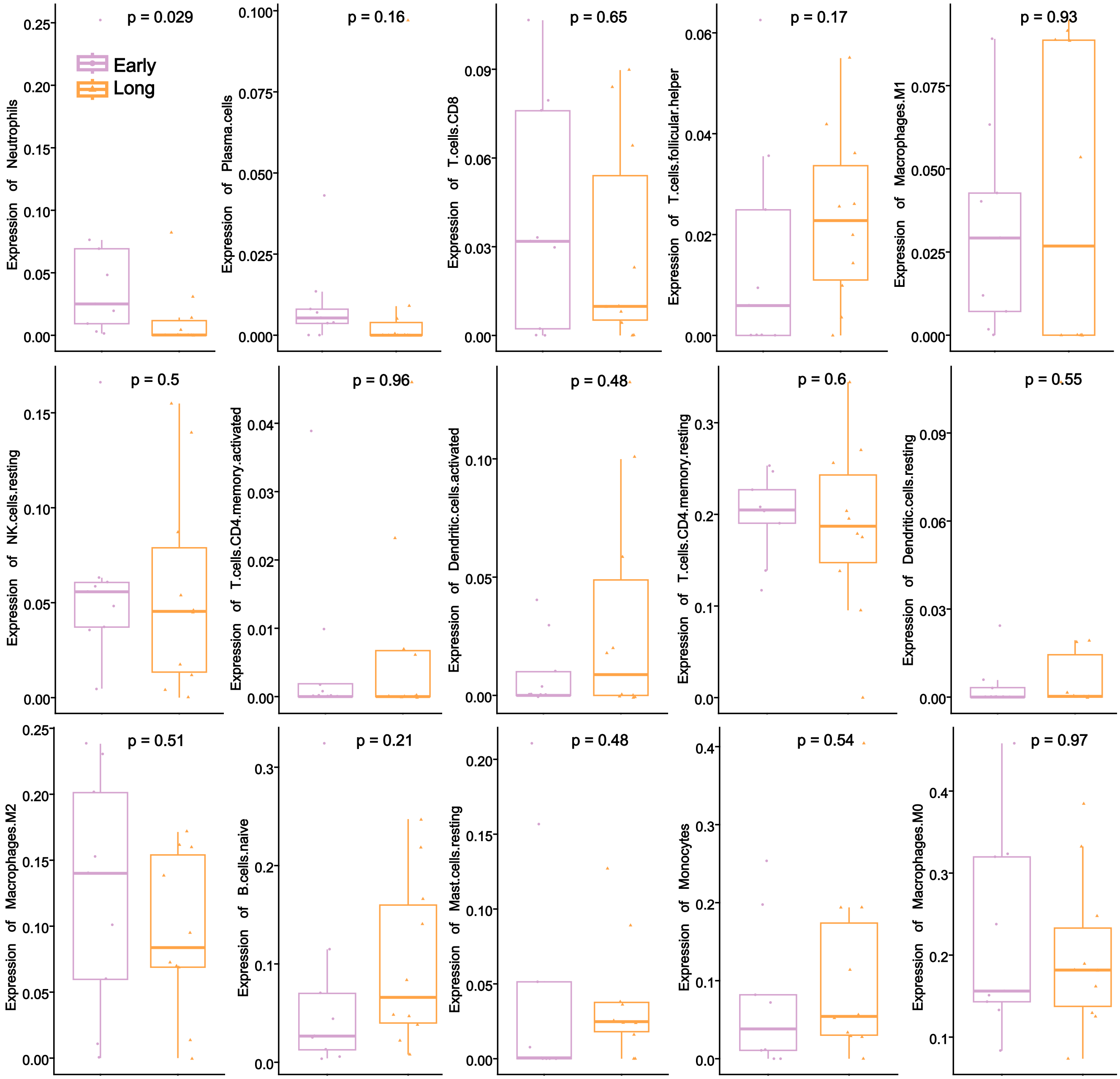

Figure. S13.

Comparison of each immune cell infiltration abundance between early progression and long benefit groups.

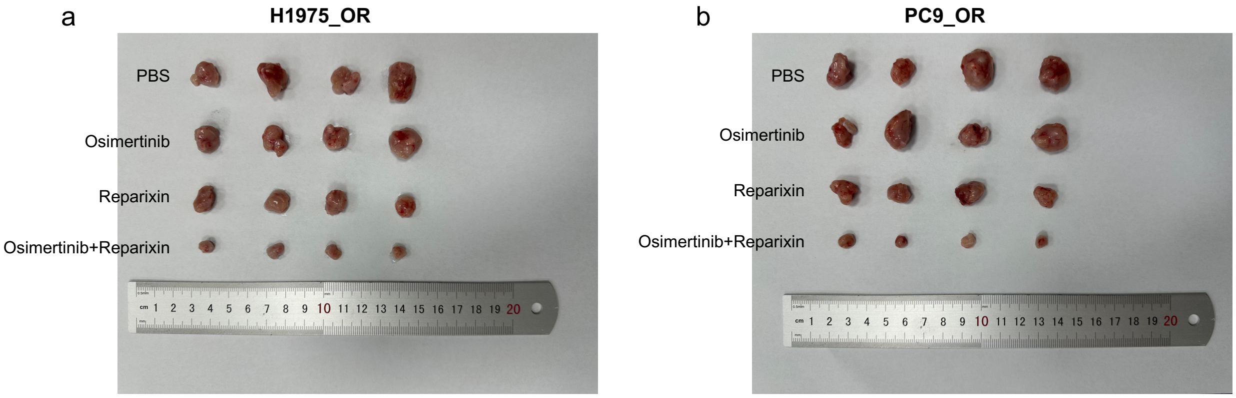

Figure. S14.

Original figures of tumor mass in different treatment groups. (**a**) H1975_OR cell line derived xenografts. (**b**) PC9_OR cell line derived xenografts.

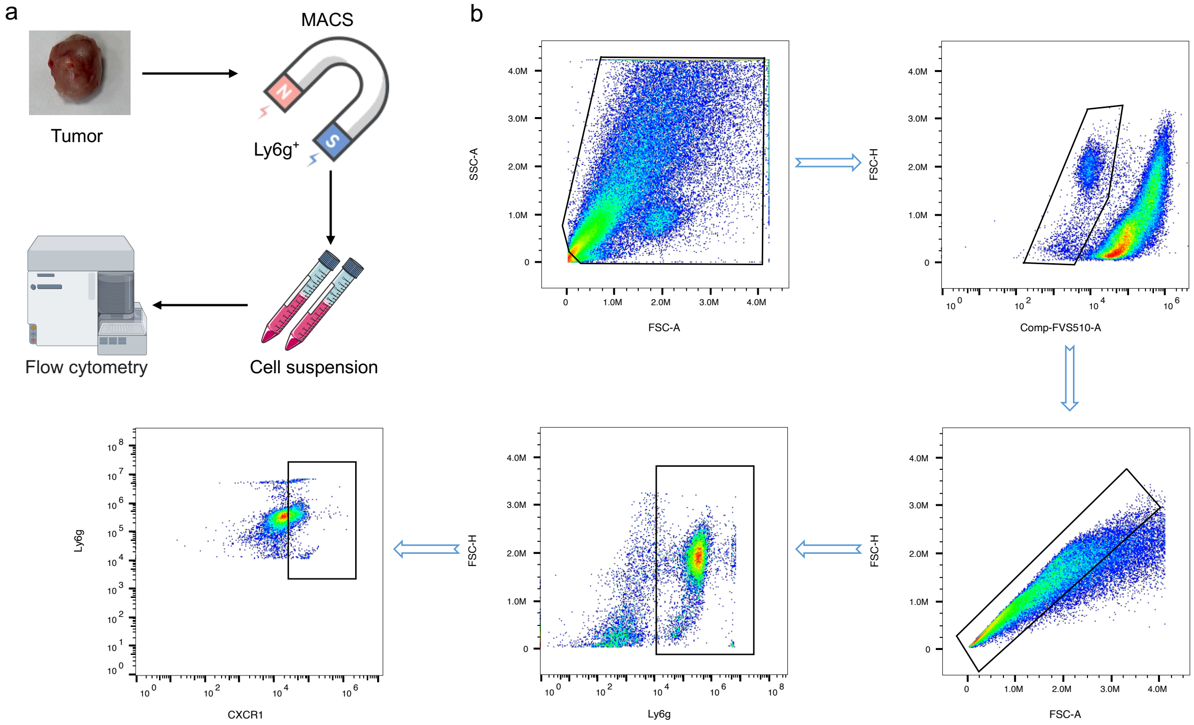

Figure. S15.

(**a**) Neutrophils in tumor were sorted with microbeads (the graphical model figure was created using Figdraw). (**b**) Flow cytometry gating strategy diagram.

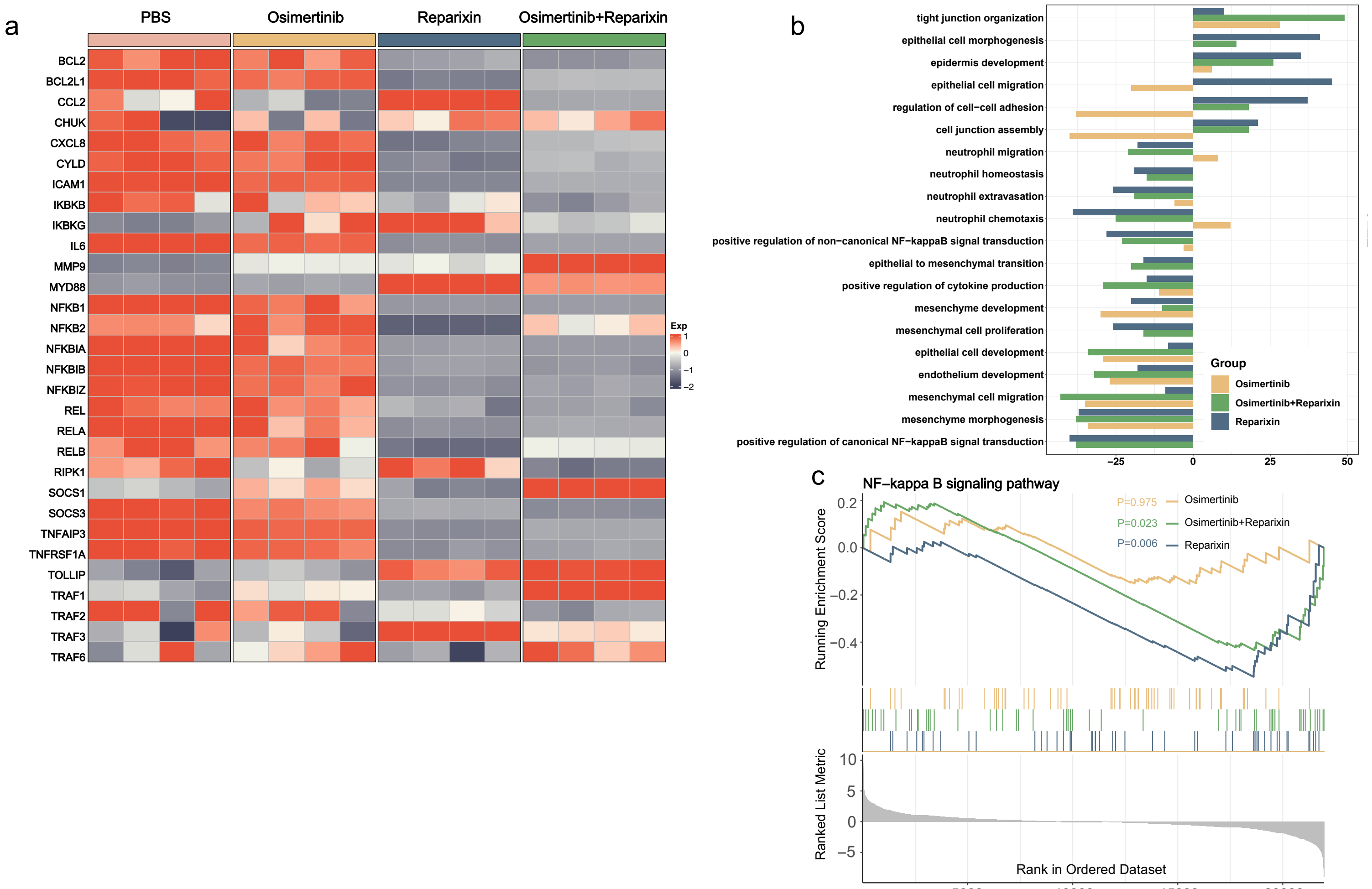

Figure. S16.

(**a**) Heatmap showing the expression profiles of NF-κB related genes. (**b**) GO enrichment analysis of differently expressed genes. (**c**) GSEA enrichment analysis of NF-κB signaling pathway in different treated groups.

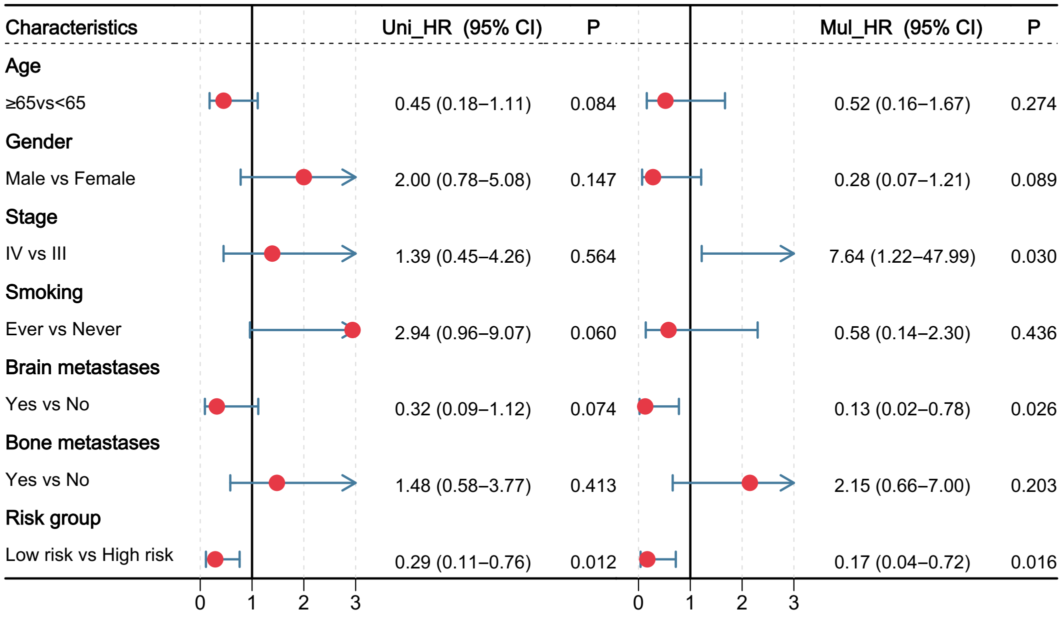

Figure. S17.

The univariate and multivariate Cox regression analysis was performed to analyze the impact of baseline neutrophil infiltration on the progression free survival. High-risk group was defined as pretreatment tumors samples with ≥ median CXCR1^{+} neutrophil infiltration level. Low-risk group was defined as pretreatment tumors samples with < median infiltration level.

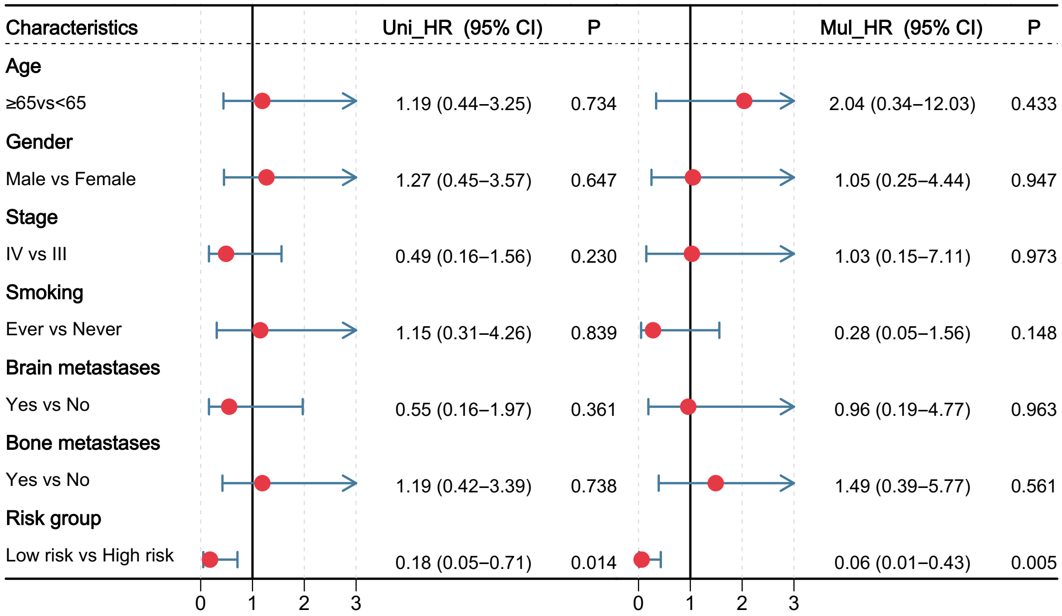

Figure. S18.

The univariate and multivariate Cox regression analysis was performed to analyze the impact of baseline neutrophil infiltration on the overall survival. High-risk group was defined as pretreatment tumors samples with ≥ median CXCR1^{+} neutrophil infiltration level. Low-risk group was defined as pretreatment tumors samples with < median infiltration level.

Table S1.

Baseline demographic and characteristics of all included and analyzed patients.

Characteristics	Overall (n=25)	Analyzed (n=17)
Age, median (range)	64 (43-78)	63 (43-76)
<65	15 (60.0)	10 (58.8)
≥65	10 (40.0)	7 (41.2)
Gender (%)		
Female	10 (40.0)	8 (47.1)
Male	15 (60.0)	9 (52.9)
Smoking status (%)		
Never	17 (68.0)	13 (76.5)
Ever	8 (32.0)	4 (23.5)
Pathological types (%)		
Adenocarcinoma	25 (100.0)	17 (100.0)
Others	0 (0.0)	0 (0.0)
EGFR mutation type (%)		
T790M	25 (100.0)	17 (100.0)
Exon 19 deletion	13 (52.0)	11 (64.7)
L858R	9 (36.0)	6 (35.3)
Others	2 (8.0)	0 (0.0)
Disease stage (%)		
III	4 (16.0)	2 (11.8)
IV	21 (84.0)	15 (88.2)
Brain metastases (%)		
No	18 (72.0)	14 (82.4)
Yes	7 (28.0)	3 (17.6)
Bone metastases (%)		
No	17 (68.0)	11 (64.7)
Yes	8 (32.0)	6 (35.3)

Table S2.

Summary of clinicopathological parameters of 22 patients in scRNA sequencing cohort.

Sample ID	Tissue sample	Age	Sex	Smoking history	Histology	Gene mutation	Resistant sample	3rd EGFR-TKI
P1	Lung	56	Male	Yes	Lung adenocarcinoma	T790M/19del	No	Osimertinib
P2	Lung	70	Female	No	Lung adenocarcinoma	T790M/L858R	No	Osimertinib
P3	Lung	68	Male	No	Lung adenocarcinoma	T790M/L858R	Yes	Osimertinib
P4	Lung	61	Female	No	Lung adenocarcinoma	T790M/19del	No	Almonertinib
P5	Lung	62	Male	No	Lung adenocarcinoma	T790M/19del	No	Osimertinib
P6	Lung	59	Male	No	Lung adenocarcinoma	T790M/L858R	Yes	Osimertinib
P7	Lung	67	Female	No	Lung adenocarcinoma	T790M/L858R	No	Furmonertinib
P8	Lung	55	Female	No	Lung adenocarcinoma	T790M/19del	Yes	Osimertinib
P9	Lung	49	Female	No	Lung adenocarcinoma	T790M/19del	No	Furmonertinib
P10	Lung	60	Male	Yes	Lung adenocarcinoma	T790M/19del	Yes	Osimertinib
P11	Lung	65	Female	No	Lung adenocarcinoma	T790M/19del	Yes	Osimertinib
P12	Lung	48	Female	No	Lung adenocarcinoma	T790M/L858R	No	Osimertinib
P13	Lung	66	Female	No	Lung adenocarcinoma	T790M/L858R	No	Almonertinib
P14	Lung	71	Male	Yes	Lung adenocarcinoma	T790M/19del	Yes	Osimertinib
P15	Lung	63	Female	No	Lung adenocarcinoma	T790M/19del	Yes	Osimertinib
P16	Lung	66	Female	No	Lung adenocarcinoma	T790M/L858R	No	Furmonertinib
P17	Lung	57	Female	No	Lung adenocarcinoma	T790M/L858R	Yes	Osimertinib
P18	Lung	58	Male	Yes	Lung adenocarcinoma	T790M/L858R	Yes	Osimertinib
P19	Lung	64	Female	No	Lung adenocarcinoma	T790M/19del	Yes	Almonertinib
P20	Lung	45	Male	No	Lung adenocarcinoma	T790M/19del	No	Osimertinib
P21	Lung	73	Male	Yes	Lung adenocarcinoma	T790M/19del	Yes	Osimertinib
P22	Lung	72	Female	Yes	Lung adenocarcinoma	T790M/L858R	Yes	Furmonertinib

Movie S1.
Intravital Imaging of $CXCR1^+$ neutrophils in mouse in H1975_OR model.

Movie S2.
Intravital Imaging of $CXCR1^+$ neutrophils in mouse in PC9_OR model.
